# Pressure on Global Forests: Implications of Rising Vegetable Oils Consumption Under the EAT‐Lancet Diet

**DOI:** 10.1111/gcb.70077

**Published:** 2025-02-20

**Authors:** Maria Vincenza Chiriacò, Nikolas Galli, Melissa Latella, Maria Cristina Rulli

**Affiliations:** ^1^ Euro‐Mediterranean Center on Climate Change CMCC Foundation Lecce Italy; ^2^ Department of Civil and Environmental Engineering Politecnico di Milano Milano Italy

**Keywords:** deforestation, dietary behavior, food, GHG emissions, land use change, replacing palm oil, vegetable oils for food use

## Abstract

Global food production faces significant challenges, acting as a primary driver of land use change, biodiversity loss, and greenhouse gas (GHG) emissions, while a significant part of the world's population still struggles with food security and nutrition. In response, the EAT–Lancet Commission has proposed a healthy and sustainable planetary diet aimed at reducing resource‐intensive foods like meat, starchy vegetables, and eggs, while suggesting a 67% increase in global per capita unsaturated oil consumption (e.g., soybean, sunflower, rapeseed) and the maintenance of the current palm oil intake. Using a spatially explicit land allocation algorithm, we assess how future global food oil demand for the expected 9.2 billion people by 2050 might impact forests and other carbon‐rich ecosystems, along with associated land use change GHG emissions. We also evaluate the potential consequences of substituting palm oil with other vegetable oils, noting their different health and environmental implications. Results show that the projected 74% rise in global vegetable oil production for food by 2050 would require 317 million hectares of land—a 68% increase compared to maintaining current consumption. This could escalate pressure on forests and threaten global food security, potentially causing 115–120 million hectares of deforestation and 1163–1210 Mt. CO_2_ per year of GHG emissions from land use change, an 87% rise compared to maintaining current consumption rates. However, the EAT‐Lancet diet foresees a reduction in other high‐impact foods, potentially freeing other lands and reducing overall projected global food GHG emissions. Another relevant finding reveals that replacing palm oil with other oils would result in increasing land needs, up to 385 million hectares with a potential 148 million hectares of deforestation, and GHG emissions, up to 1525 Mt. CO_2_ per year, thus not representing a conclusive and viable solution towards sustainability. Instead, along with the growing importance of certification schemes for sustainable and deforestation‐free food supply chains, ensuring sustainable production of all vegetable oils emerges as a critical strategy to prevent the conversion of biodiverse and carbon‐rich lands.

## Graphical abstract

The projected rise in global per capita vegetable oil consumption for the expected 9.2 billion people by 2050 would increase land needs. This could escalate pressure on forests and threaten global food security, potentially causing 115–120 million hectares of deforestation and 1163–1210 Mt. CO_2_ per year of GHG emissions from land use change, an 87% rise compared to maintaining current consumption rates. Replacing palm oil with other oils would result in a further increase of land use and GHG emissions from land use change, thus not representing a conclusive and viable solution towards sustainability. Along with the growing importance of certification schemes for sustainable and deforestation‐free food supply chains, ensuring sustainable production of all vegetable oils emerges as a critical strategy to prevent the conversion of biodiverse and carbon‐rich lands.

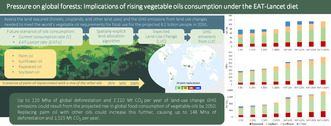

## 1 Introduction

Food production stands as the largest global driver of land use change (Ramankutty et al. 2018; Song et al. 2018; Aznar‐Sánchez et al. 2019; Bombelli et al. 2019; Galli et al. 2023), primarily causing forest clearance and biomass burning, along with the loss of many areas of natural grasslands, savanna, cerrado, and pampa biomes, with consequent biodiversity loss and greenhouse gas (GHG) emissions (Crippa et al. 2021), ultimately undermining critical Earth system processes (Stehfest et al. 2019; Winkler et al. 2021). Besides, a significant portion of the world's population suffers from inadequate nutrition, with widespread poor or unhealthy diets that pose risks of morbidity and mortality to many individuals, causing undernutrition, micronutrient deficiency (FAO 2022), and various diet‐related non‐communicable diseases, including overweight or obesity (Swinburn et al. 2019; Ng et al. 2014), cardiovascular disease, and type II diabetes (Lim et al. 2012). Therefore, providing a growing global population with healthy diets from sustainable food systems is an urgent challenge.

Against this challenging backdrop, the EAT‐Lancet Commission has introduced a global benchmark diet aimed at guiding the transition towards healthier and more sustainable dietary patterns (Willett et al. 2019). Among the ranges of nutrients set for an ideal energy intake of 2500 kcal/day to ensure human health and safe planetary boundaries, the EAT‐Lancet Commission suggests a specific global average per capita intake of 6.8 g/day as ideal palm oil consumption, corresponding to 2.5 kg/capita/year, which is roughly in line with the current intake of 2.6 kg/capita/year palm and palm kernel oil (FAOSTAT 2023), although this value may be underestimated due to unaccounted subsistence‐level palm oil consumption in Africa (Carrere 2010; Descals et al. 2024), and 40 g/day as global average per capita ideal intake of unsaturated oils, corresponding to 14.6 kg/capita/year, which means to increase by 67% the current consumption of 8.7 kg/capita/year (FAOSTAT 2023).

The global population is projected to reach 9.2 billion people in 2050, according to the Shared Socioeconomic Pathway (SSP) 2 for moderate growth scenario (IPCC 2021). This, combined with the suggested increase of per capita unsaturated oil consumption, would necessitate a rise in the production of vegetable oils and an expansion of the area required for their cultivation, potentially exacerbating the current pressure of vegetable oils' production on forests and other critical ecosystems.

Today, approximately 330 million hectares of land worldwide are dedicated to oil crop cultivation, and the global production of vegetable oils exceeds 200 million tons annually, with nearly 40% being utilized for direct human consumption (FAOSTAT 2023). The most produced vegetable oil is palm oil, with more than 73.5 Mt. annually, in addition to approximately 8 Mt. of palm kernel oil, followed by soybean oil with nearly 58 Mt., rapeseed oil with almost 25 Mt., and sunflower oil producing nearly 20 Mt. each year (based on 2018–2020 data from FAOSTAT 2023). Several of these oil crops, especially oil palm plantations and soy cultivation, along with other commodities like beef, timber products, coffee, rubber, cocoa, and sugar, play a significant role in driving global land use changes (Pendrill, Persson, Godar, and Kastner 2019; Pendrill, Persson, Godar, Kastner, et al. 2019; Goldman et al. 2020), including deforestation, which affects around 10.2 million hectares annually (averaging from 2015 to 2020; FAO‐FRA 2020). Global oil production accounts for almost 20% of what is referred to as commodity‐driven deforestation (Pendrill, Persson, Godar, and Kastner 2019).

The expected expansion of oilseed crops to meet the future global food requirements is likely to further exacerbate the risk for the global forests, which cover approximately 31% of the Earth's land area, spanning roughly 4.06 billion hectares. Forests serve a multitude of crucial ecological roles, hosting a substantial portion of the world's terrestrial biodiversity (Moomaw et al. 2020). Additionally, they are pivotal in climate regulation, enhancing soil, air, and water quality, and are essential in the carbon cycle and climate change mitigation (Friedlingstein et al. 2022). Global forests store approximately 1085 gigatonnes of carbon (FAOSTAT 2023) and remove approximately 7.6 ± 49 Gt CO_2_ from the atmosphere annually (Harris et al. 2021), being therefore a crucial mechanism for achieving the climate neutrality objectives outlined in the Paris Agreement, aimed at limiting the global temperature increase to well below 2°C above preindustrial levels.

Notably, palm oil has come under intense scrutiny within the realm of agricultural commodities due to the well‐established connection between the expansion of oil palm cultivations and the alarming trend of depletion of tropical forests observed in recent decades (Vijay et al. 2016; Teng et al. 2020). In fact, over the past few decades, the conversion of substantial portions of tropical forests and the drainage of natural peatlands for the establishment of oil palm plantations have led to the loss of crucial carbon reservoirs, with consequent significant GHG emissions (Cooper et al. 2020; Dhandapani and Evers 2020) and the destruction of vital natural habitats resulting in biodiversity losses and triggering soil erosion phenomena (Dradjat 2012; Schrier‐Uijl et al. 2013; Khatun et al. 2017; Pacheco et al. 2017; Vijay et al. 2016; Austin et al. 2017; Carlson et al. 2018; Rulli et al. 2019; Lee et al. 2020).

The growing attention to this issue has led to varied responses regarding palm oil use, especially within the food sector, influencing both supply and demand dynamics (Vergura et al. 2019; Chiriacò et al. 2022; Savarese et al. 2022). The interest in potential substitutes for palm oil, notably soybean, rapeseed, and sunflower oils, has increased due to their availability, pricing, and producing conditions, making them viable alternatives, especially for food applications (Parsons et al. 2020; Busch et al. 2022; Gaveau et al. 2022). Simultaneously, a significant segment in the agri‐food sector has committed to zero‐deforestation pledges (Austin et al. 2017), opting for sustainably certified palm oil production and/or usage, ensuring protection of high carbon‐content ecosystems like forests and peatlands, and refraining from converting swamp forests, typically prone to intentional fires (Carlson et al. 2018; Chiriacò et al. 2022).

Given the finite nature of land resources (Hoekstra and Wiedmann 2014) and the intense competition for various land uses (IPCC 2019a), each with differing contributions to climate change (Popp et al. 2014; Hasegawa and Matsuoka 2015), it becomes crucial to understand the feasibility (Cirigliano et al. 2017) and associated environmental costs, particularly in terms of GHG emissions, of meeting the world's 2050 food oil requirements.

To this aim, this study assesses the likely future global distribution of the four main oil crops (oil palm, soybean, sunflower, rapeseed), the potential land use changes (LUC), including deforestation, and the resulting GHG emissions that could occur to satisfy the world's food oils requirement in 2050. Additionally, we evaluate the potential impacts of substituting palm oil in food applications with other edible vegetable oils, hypothesizing four palm oil replacement scenarios, where 0%, 25%, 50%, and 100% of palm oil is substituted by a mix of the three other oils.

## 2 Materials and Methods

### 2.1 Building Future Scenarios of Oil Consumption

To assess the global amount of palm, soybean, sunflower, and rapeseed oil needed to meet the world's food requirements in 2050, two consumption scenarios were considered (Table 1), based on (i) the current global average per capita consumption rate of each oil derived by FAOSTAT (2023) as the average of the global consumption per person for the years 2018–2020 and (ii) the average per capita intake of palm oil and unsaturated oils as suggested by the EAT‐Lancet global benchmark diet (Willett et al. 2019). Both the consumption scenarios were built considering a projected global population growth to 9187 million people in 2050 as expected under the SSP2 (IPCC 2021). Since the EAT‐Lancet average per capita intake of palm oil does not differentiate between palm and palm kernel oils, their respective amounts in the EAT‐Lancet consumption scenario were determined according to their current share (last column in Table S1). Likewise, the intake of unsaturated oils was broken down into three vegetable oils, which are sunflower, soyabean, and rapeseed, according to their current share.

**TABLE 1:** The oil amount for food use in 2050 with 9.2 billion world inhabitants (SSP2) is based on the current consumption rates as an average of 2018–2020 (FAOSTAT 2023) and EAT‐Lancet oil rates.

Consumption scenarios			Current consumption rate				EAT‐Lancet rate			
Palm oil replacement scenarios			0%	25%	50%	100%	0%	25%	50%	100%
Oil	Global avg. consumption rate (kg/capita per year)		Expected food oil production in 2050 (Mt)							
	Current	EAT‐Lancet								
Palm	2.4	2.5	22	16.5	11	0.0	21.1	15.8	10.6	0.0
Palm kernel	0.19		1.8	1.4	0.9	0.0	1.7	0.4	0.8	0.0
**Total palm oil**			23.8	17.9	11.9	0.0	22.8	16.2	11.4	0.0
Rapeseed	1.24	2.08	11.4	12.7	13.9	16.4	19.1	20.5	21.5	23.9
Soyabean	3.14	5.25	28.9	32	35.2	41.5	48.2	51.7	54.3	60.3
Sunflower	1.55	2.60	14.3	15.8	17.4	20.5	23.9	25.6	26.8	29.8
**Total main unsaturated oils**			54.6	60.5	66.5	78.4	91.2	97.8	102.6	114
**Total palm and unsaturated oils**			78.4	78.4	78.4	78.4	114	114	114	114
Other oils	2.79	4.67	25.7	25.7	25.7	25.7	42.9	42.9	42.9	42.9
**Total**	11.33	17.10	104	104	104	104	157	157	157	157

Furthermore, the four palm oil replacement scenarios were designed assuming substitutions ranging from 0%, 25%, 50% to 100% of palm and palm kernel oil for food uses employing a blend of the three alternative vegetable oils. The quantities of palm and palm kernel oil slated for substitution under the different replacement scenarios were allocated to the three alternative oils based on their respective shares of global production (last column in Table S1).

### 2.2 Land Allocation Algorithm

An ad hoc land allocation algorithm was developed to determine the potential land use change that can occur to meet the world's vegetable oils food requirements in 2050 under the two consumption scenarios and with the different palm oil replacement options. The land use change algorithm relies on four sets of criteria: inclusion, exclusion, attribution, priority.

Inclusion criteria define areas that would likely be affected by oilseed crop cultivation. These include areas that are currently bare lands, grasslands, shrublands, areas harvested with rainfed and irrigated annual crops, areas harvested with rainfed and irrigated perennial crops, and forests. Annual and perennial crop‐specific areas are provided by SPAM2010 (Yu et al. 2020) as a 5‐arcmin resolution raster, with the information stored in each pixel being the extent, in hectares, of the harvested area within the pixel. The other areas are retrieved from the ESA‐CCI land cover maps (Bontemps et al. 2016) and reprocessed to match the characteristics of the harvested area maps. Moreover, peatlands were also included as areas potentially affected and were derived from the Peat‐ML dataset (Melton et al. 2021).

Exclusion criteria define areas that cannot be used for the oilseed crop's cultivation, namely (i) the areas retrieved from the ESA‐CCI land cover maps (Bontemps et al. 2016) other than those included according to the inclusion criteria (i.e., urban areas, water bodies, wetlands) and (ii) areas with low suitability for the target oilseed crop. The crop‐specific suitability maps, each with 8 classes (1. very high, 2. high, 3. good, 4. medium, 5. moderate, 6. marginal, 7. very marginal, 8. not suitable), are obtained for the 4.5 Representative Concentration Pathway (RCP) from the Global Agro‐Ecological Zones Data Portal (FAO 2021). In particular, a suitability class lower or equal to “6. marginal” is considered a sufficient condition for the exclusion of the pixel.

Attribution criteria assign each pixel to a specific oil crop, selecting the crop most suitable for the pixel and, if equal the crop with the highest attainable crop yield. The crop‐specific suitability maps as well as the crop‐specific attainable yield, both for the 4.5 RCP, are obtained from the Global Agro‐Ecological Zones Data Portal (FAO 2021).

Finally, priority criteria define in which pixels the algorithm must look first for possible area available for cultivation, meaning they determine the spatial dynamic of the future oil crops distribution. These criteria are, in descending order of priority: (i) higher suitability to the assigned oil crop, (ii) higher attainable yield, or, only for oil palm cultivation, closeness to areas already harvested with oil palm (i.e., share of the pixel area already occupied by oil palm plantations). In fact, specifically for oil palm, which is the sole perennial woody oil crop among the oil crops analyzed, it would be most cost‐effective to plan future cultivation both in areas highly suitable and with the highest yield or in highly suitable areas already hosting oil palm plantations or close to them, assuming that the 25‐year long oil palm crop cycle would make unlikely changes towards land uses other than oil palm.

To support discussion, each pixel was also attributed to a country, based on the Database of Global Administrative Areas (GADM 2022), and to a bioclimatic region according to the Thermal Climate Zones of the World dataset included in the FAO Food Insecurity, Poverty, and Environment Global GIS Database (FGGD) (Van Velthuizen 2007).

After removing pixels based on exclusion criteria and assigning each included pixel to an oil crop, the algorithm sorts the pixels in the inclusion criteria maps for each oil crop according to their order of priority and then multiplies the available area in each pixel by the crop‐specific yield attainable in that pixel to determine and then cumulate the potential oil production. To calculate oil production (see Supporting Information, Equations 1–4), we utilized attainable crop yields expressed as seed yield (FAO 2021) and applied the oil/seed ratio derived from literature sources. Specifically, the ratios used are 0.20 for soybean (Dijkstra 2016), 0.35 for rapeseed (Wei et al. 2008), and for sunflower (Le Clef and Kemper 2015), except for palm oil, for which attainable oil yields are directly provided. Furthermore, in the case of soybean, from which products other than oil are also commonly produced (e.g., flour, lecithin, soymilk, beans, etc.), we considered the global ratio of seeds allocated to oil production over the total seed yield (84%), as determined by FAOSTAT (2023). Additionally, the algorithm scales for each oil crop the total attainable oil production in each pixel based on the portion of oil designated for food use. This scaling is determined according to the global average share of oil for food use over the total oil production, as provided by FAOSTAT (2023). The assigned percentages are 25% for palm oil, 38% for rapeseed oil, 41% for soybean oil, and 61% for sunflower oil (fifth column in Table S1). In this way, the projection of future areas affected by oil crop cultivation to meet the global expected oil needs in 2050 represents the areas actually needed for oil use in food, taking into account also the possible uses other than food, such as biodiesel, feed, cosmetics, etc., assuming the current distribution among uses remains constant in the future.

Once the cumulative sums of potential oil productions in the assigned areas are computed, they are compared with the required oil amount in the two consumption scenarios and in the different palm oil replacement scenarios (Table 1). This analysis aims to derive the minimum set of characteristics that pixels must possess for each oil crop cultivation under each scenario, according to the priority criteria, that is, (i) suitability to the crop, (ii) yield, or, exclusively for oil palm, the portion of the pixel area already under oil palm cultivation, to detect the eligible pixels.

### 2.3 Assessing Land Use Change GHG Emissions

The potential GHG emissions from LUC occurring for the oil crops establishment are evaluated with respect to the current land use by gauging potential losses in carbon stock from areas with high carbon content, such as forests, peatlands, and perennial croplands (Kotowska et al. 2015).

Forest ecosystems as well as perennial crop systems represent important carbon stocks (Friedlingstein et al. 2022) that store carbon in pools including above‐ and below‐ground living biomass, dead wood, litter, and soil (IPCC 2006, 2019b). The total amount of carbon stored and its share in each pool vary according to the type of forest or orchard, its management, and its geographical location (IPCC 2019b). When a land use change occurs on those systems towards an annual oilseed crop, whether through fires, uprooting, or slash and burning, the total amount of carbon stored is considered as immediately oxidized and released into the atmosphere, thus resulting in GHG emissions (IPCC 2006, 2019b). Also, peatlands represent important soil carbon stocks, which, although occupying only 3% of the global land area, contain about 25% of global soil carbon (Lourenco et al. 2023). They are organic soils in wetland ecosystems in which waterlogged conditions prevent plant material from fully decomposing, thus reaching an organic content of > 35%. When peatlands are drained for agricultural purposes, the water table is artificially lowered, and organic soils readily decompose as the conditions become aerobic, causing the oxidation of the high amount of carbon stored and consequent CO_2_ emissions (Hergoualc'h et al. 2020; McCalmont et al. 2021). Such emissions continue for decades and even centuries after the drainage (Conchedda and Tubiello 2020), besides other impacts such as the high risk of exposed peat to burning and subsidence that can cause flooding (Hooijer et al. 2012; Matysek et al. 2018; Cooper et al. 2020).

In the case of forests converted to oil crops, potential emissions from the land use change were assessed considering the country‐specific carbon stock loss from the above‐ and below‐ground biomass of forest areas potentially affected by oil crop cultivation, as derived by the Global Forest Resource Assessment of the Food and Agriculture Organization of the United Nations (FAO‐FRA 2020). According to IPCC (2019b), a land use change results in most cases in a loss of soil carbon for some years following conversion, regardless of soil type (i.e., mineral or organic). However, in the absence of reliable data, the soil carbon pool was not considered in this study, and the same applied to dead wood and litter.

In the case of perennial croplands (both irrigated and rainfed) converted to an oil crop, only the carbon stock change in above‐ground biomass was considered to estimate the GHG emissions since limited data are available on below‐ground carbon stocks (IPCC 2006, 2019b). The used values of above‐ground biomass carbon stock at maturity are equal to 63 Mg C ha^−1^ for temperate orchards, 21 Mg C ha^−1^ for tropical moist orchards, and 9 Mg C ha^−1^ for tropical dry and boreal orchards, all with a maturity cycle of 30 years (IPCC 2006). As for forest conversion, in the absence of reliable data, the soil carbon pool was not considered. Moreover, it was assumed that the carbon content in dead wood and litter of perennial croplands is negligible (IPCC 2006, 2019b).

The annual GHG emissions from forests and perennial croplands potentially replaced by oil crop cultivation were assessed, assuming that, once established after an LUC, an oil crop will be maintained in the same area for at least 25 years. Therefore, the potential annual emissions were considered as one twenty‐fifth of the total carbon stock that is lost in the year of the land conversion (IPCC 2019b), at the net of the annual carbon sink in the above‐ground biomass occurring solely in case of conversion to an oil palm plantation (Quezada et al. 2022), calculated considering a carbon stock at maturity equal to 40 Mg C ha^−1^ at 25 years of age (Khasanah et al. 2015).

When peatlands are drained for oil crop cultivation, the major emissions arise from the removal and destruction of the living biomass of the peatland ecosystem and from soils during and after the drainage (IPCC 2006, 2014). The loss of carbon in living biomass was accounted according to the methods described in the previous paragraphs, while emissions from drained soil were calculated using the method provided by IPCC (2006), which suggests using the emission factor for nutrient‐poor peatlands equal to 0.2 Mg C ha^−1^ year^−1^ for boreal countries, that of nutrient‐rich peatlands of 1.1 Mg C ha^−1^ year^−1^ for temperate countries, and the default factor of 2 Mg C ha^−1^ year^−1^ for the tropical regions.

When a land use change occurs on other land uses, such as bare areas, grasslands, shrublands, and annual croplands (both irrigated and rainfed), no carbon stock change was assessed in any carbon pool. In fact, the biomass carbon stock in bare areas is zero. Also, it is assumed to be zero in grasslands and annual croplands where the increase in biomass stocks during the year compensates for biomass losses from harvest and mortality in that same year (IPCC 2019b). The biomass carbon stock change in shrublands was not included due to a lack of specific data. Moreover, in the absence of reliable data, the carbon in the soil, dead wood, and litter was not considered in any of these land uses.

Additionally, the potential threat to global food security is assessed by considering the extent of the current annual and perennial crop‐specific areas, as outlined in SPAM2010 (Yu et al. 2020), that might be impacted by the future distribution of oil crops.

## 3 Results

### 3.1 Potential Future Distribution of Palm Oil Plantations for Food Use

**FIGURE 1:**
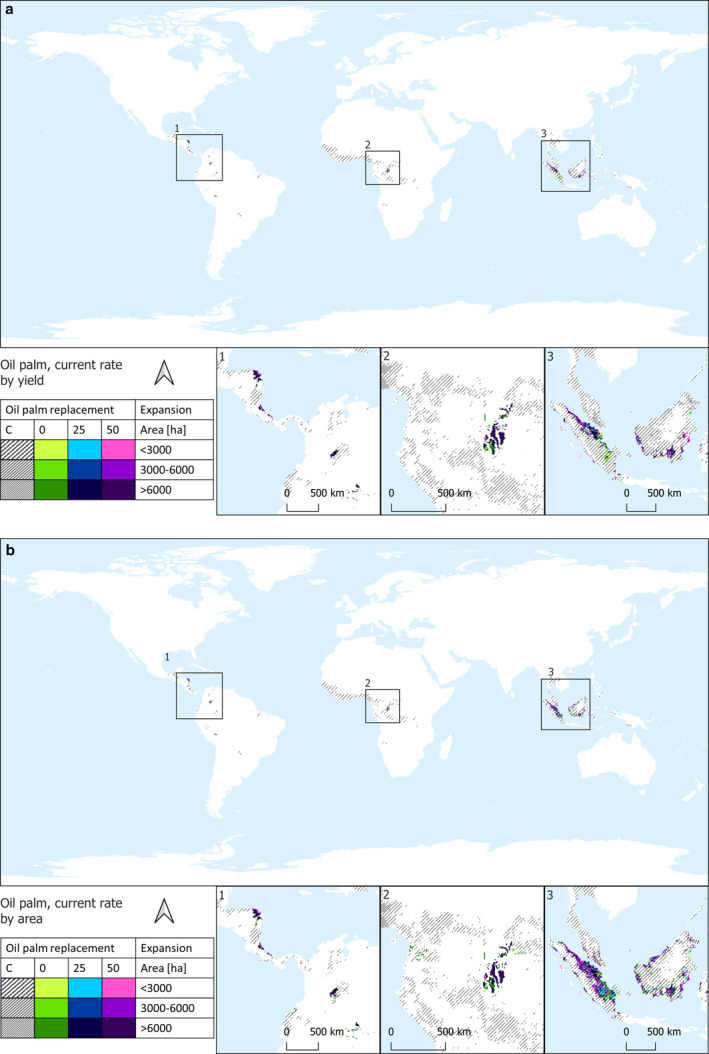
Potential future distribution of oil palm plantations for food use in 2050 under the ‘current consumption rate scenario.’ Suitable areas are designated based on the potential for higher attainable yields (a) and proximity to existing oil palm plantations (b). Grey lines indicate the current distribution, with three cover intensities (< 3, 3–6, > 6 kha). Colored pixels, with three cover intensities, indicate future suitable areas for oil palm plantations. Specifically, violet pixels indicate areas suitable to host plantations in the 50% palm oil replacement scenario; blue pixels indicate those areas suitable to complement the violet areas in the 25% palm oil replacement scenario; and green pixels indicate areas suitable to complement the violet and the green areas in the no palm oil replacement scenario.

**FIGURE 2:**
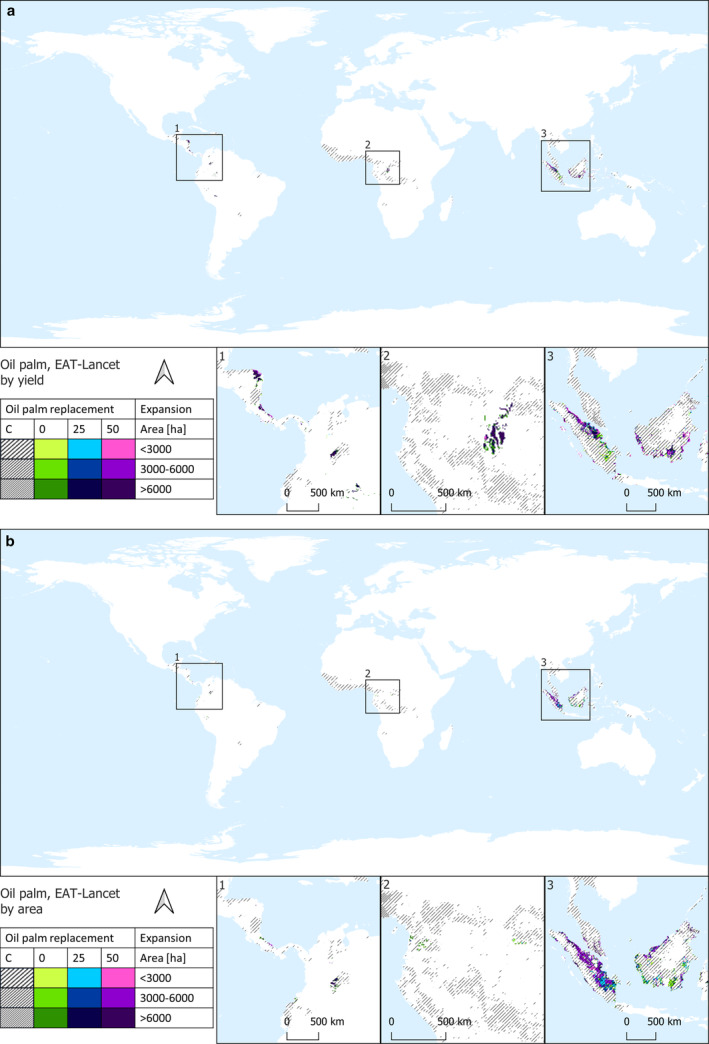
Potential future distribution of oil palm plantations for food use in 2050 under the ‘EAT‐Lancet rate scenario.’ Suitable areas are designated based on the potential for higher attainable yields (a) and proximity to existing oil palm plantations (b). Grey lines indicate the current distribution, with three cover intensities (< 3, 3–6, > 6 kha). Colored pixels, with three cover intensities, indicate future suitable areas for oil palm plantations. Specifically, violet pixels indicate areas suitable to host plantations in the 50% palm oil replacement scenario; blue pixels indicate those areas suitable to complement the violet areas in the 25% palm oil replacement scenario; and green pixels indicate areas suitable to complement the violet and the green areas in the no palm oil replacement scenario.

Results are shown through maps illustrating the potential future distribution of areas designated for oil palm plantations for food use in 2050. Figure 1 depicts suitable future areas under the ‘current consumption rate scenario’ based on the potential for higher attainable yields (Figure 1) and on the proximity to existing oil palm plantations (Figure 1b). Similarly, Figure 2 shows the potential future distribution of oil palm plantations for food use in 2050 under the ‘EAT‐Lancet rate scenario,’ prioritizing suitable areas based on higher yield (Figure 2) and proximity to existing oil palm plantations (Figure 2b). The future world's palm oil requirements for food use in 2050 show negligible variations between the two consumption scenarios (see Table 1, Figures 1, and 2). This is attributed to the fact that the EAT‐Lancet palm oil consumption of 2.5 kg/capita/year is already approximately aligned with the current intake of palm and palm kernel oil, standing at 2.6 kg/capita/year according to FAOSTAT (2023).

Projections for 2050 prioritizing areas with the potential for higher attainable yields indicate that 16.6 or 16.1 Mha of land would be necessary to meet the future global demand for 23.8 or 22.8 Mt. of palm oil under the ‘current consumption rate scenario’ and the ‘EAT‐Lancet scenario,’ respectively (Figures 1 and 2, Table 2). These land requirements decrease to 12.4 or 12 Mha in the two consumption scenarios when the food use of palm oil is replaced by 25% of other oils, resulting in 17.8 or 16.2 Mt. of palm oil in the respective scenarios. Furthermore, the land area decreases to 8.2 or 8 Mha in the consumption scenarios with a 50% replacement in palm oil food use (Table S2). Nearly 60% of these areas are concentrated in Southeast Asia, primarily in Indonesia, which includes an overlap of about 1.6 million hectares with existing oil palm plantations. Other significant contributors in this region are Malaysia, Brunei Darussalam, Papua New Guinea, and the Philippines. Approximately 18% of the identified areas are situated in Central America, spanning countries such as Colombia, Honduras, Nicaragua, Costa Rica, Panama, Venezuela, the Dominican Republic, and Guatemala, and 16% is distributed in Central Africa and Madagascar.

**TABLE 2:** Land required to meet the world's future oil requirements for food use in 2050 and potential GHG emissions from land use changes.

Oil crop	Unit	Current consumption rate				EAT‐Lancet rate			
		0%	25%	50%	100%	0%	25%	50%	100%
Rapeseed	Mha	25.8	29.1	32.4	39.5	47.6	52.0	55.4	63.4
Soybean		103.5	115.7	128.4	153.3	181.3	195.0	206.2	231.2
Sunflower		41.8	46.5	51.4	61.0	71.7	80.4	81.0	90.9
**Total unsaturated oils**		171	191	212	254	301	327	343	385
Palm (by yield)		16.6	12.4	8.2	0.0	16.1	12.0	8.0	0.0
**Total (by yield)**		188	204	220	254	317	339	351	385
Palm (by area)		17.5	13.0	8.7	—	16.7	12.4	8.3	—
**Total (by area)**		189	204	221	254	317	340	351	385
Rapeseed	MtCO_2_ yr^−1^	118	139	161	206	257	283	303	348
Soybean		334	375	423	524	668	747	810	947
Sunflower		97	107	117	142	172	199	201	230
Palm (by yield)		116	87	54	—	112	84	53	—
**Total (by yield)**		666	708	755	872	1210	1314	1366	1525
Palm (by area)		71	41	21	—	65	38	20	—
**Total (by area)**		621	662	722	872	1163	1268	1333	1525

Similar results, in terms of total area, are observed when considering the world's palm oil food requirements in 2050 by prioritizing suitable areas per closeness to areas already harvested with oil palm (Figures 1b and 2b, Table 2). In total, 17.5 or 16.7 ha of land would be necessary under the current consumption rate scenario and the EAT‐Lancet scenario, respectively, to meet the future demand for 23.8 or 22.8 Mt. palm oil in food consumption. This land requirement decreases to 13 or 12.4 Mha in the two consumption scenarios when the food use of palm oil is replaced by 25% of other oils. Additionally, it further reduces to 8.7 or 8.3 Mha in the two consumption scenarios when there is a 50% replacement in palm oil food use (Table S2). However, the geographical distribution pattern in this case distinctly indicates that more than 90% of future palm oil plantations for food use would likely be concentrated in Indonesia and Malaysia, where approximately 4 Mha already host existing oil palm plantations. This potential future distribution of oil palm plantations, prioritizing suitable areas based on proximity to existing plantations, appears to be the most efficient in terms of land use. In fact, by harnessing about 4 Mha of land already allocated to oil palm (Table S4), it has the potential to impact fewer forested areas, that is, 1.7–6.5 Mha (Table S2), compared to the potential oil palm distribution based on higher yield criteria, which could affect 5–10.9 Mha of global forests (Table S2).

**FIGURE 3:**
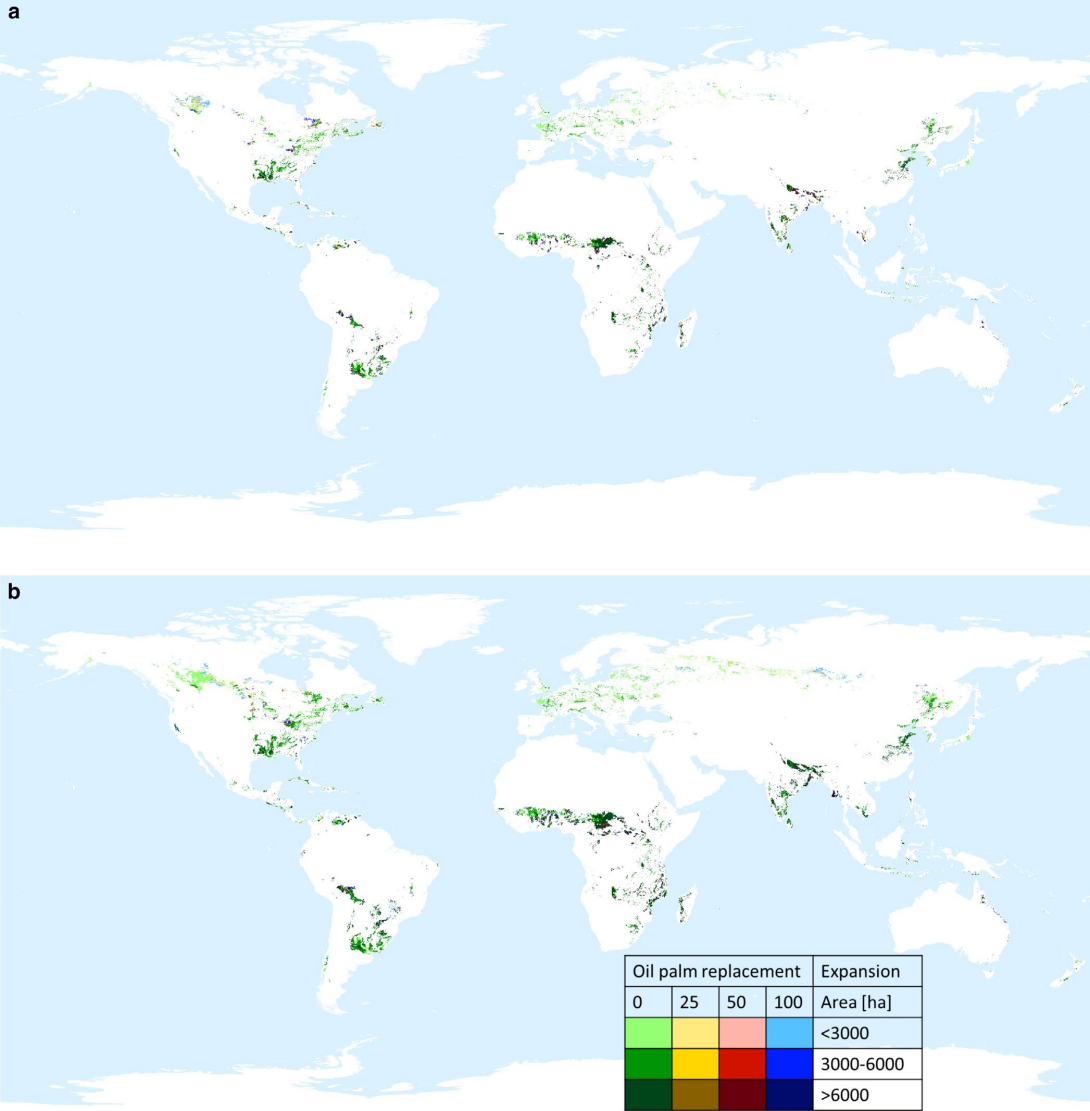
Potential future distribution of cumulative areas of oil crops (rapeseed, sunflower, soybean) for food use in 2050 under the ‘current consumption rate scenario’ (a) and ‘EAT‐Lancet rate scenario’ (b). Green pixels indicate areas suitable to cover the no palm oil replacement scenario; yellow pixels indicate areas suitable to complement the green areas to satisfy the 25% palm oil replacement scenario; red pixels indicate areas suitable to complement green and yellow areas to satisfy the 50% palm oil replacement scenario; blue pixels indicate areas suitable to complement green, yellow, and red areas to satisfy the 100% palm oil replacement scenario. Each colored pixel is represented with three cover intensities (< 3, 3–6, > 6 kha).

Figure 3 showcases the maps of the potential future distribution of areas allocated for unsaturated oils production for food use in 2050, under the ‘current consumption rate scenario’ (Figure 3) and the ‘EAT‐Lancet rate scenario’ (Figure 3b). The results show that to meet the 54.6 and 91.2 Mt. of unsaturated oil food demand in 2050 (Table 1) in the current consumption scenario and the EAT‐Lancet scenario, respectively, an estimated 171 and 301 Mha of land is required. These areas expand to 191–254 and 327–385 Mha, in the two consumption scenarios if 25% to 100% palm oil is replaced by unsaturated oils (Table 2).

Specifically, to meet the rapeseed oil food demand in 2050 based on current consumption rates, an estimated 25.8 Mha of land is required. This area expands to 29.1–39.5 Mha under the 25% to 100% palm oil replacement scenarios. Evenly, if we project the EAT‐Lancet consumption rate to 2050, the required land increases to 47.6 Mha, with a further rise to 52.0–63.4 Mha under the palm oil replacement scenarios (Table S2). The identified land is predominantly located in Canada, the USA, France, and various European countries, including Poland, the UK, Belarus, Ukraine, Germany, Italy, and Chile (see Figure S1). This could potentially impact 13.1–23.7 Mha of land currently covered by forests under the scenario with the current consumption rate and 30.0–41.6 Mha under the EAT‐Lancet consumption scenario. Additionally, 8.3–10.3 and 11.7–15.0 Mha, currently occupied by annual crops, in the two consumption scenarios, respectively, result in being suitable for rapeseed cultivation in 2050, posing also another important issue related to a potential risk for food security (Table S2).

Similarly, to meet the projected sunflower oil food demand by 2050 based on current consumption rates, about 41.8 Mha of land would be necessary. This figure expands to 46.5–61.0 Mha under the 25% to 100% palm oil replacement scenarios. Similarly, under the EAT‐Lancet scenario, the required land increases to 71.7 Mha, with a further rise to 80.4–90.9 Mha under the palm oil replacement scenarios (Table S2). The identified land is primarily situated in the USA and Canada, Argentina and Uruguay, China, Germany, Ukraine, the Russian Federation, and South Africa (refer to Figure S2). Although the total required area is almost twice that of rapeseed, the potentially affected land currently covered by forests is sensibly lower, amounting to 10.6–15.7 Mha under the scenario with the current consumption rate and 19.3–25.8 Mha under the EAT‐Lancet consumption scenario. Contrarywise, a greater extent of grasslands, reaching up to 12.8 Mha, is affected compared to the rapeseed extension. Furthermore, the concern regarding food security could also be relevant for sunflower oil production, since 23.7–34.1 and 39.4–48.6 Mha currently allocated to annual crops, in the two consumption scenarios, respectively, are projected to be deemed suitable in 2050.

Soybean oil production to meet food demand in 2050 would require 103.5 Mha based on the current consumption rates. This area expands to 115.7–153.3 Mha under the palm oil replacement scenarios. Similarly, under the EAT‐Lancet scenario, the needed land increases to 181.3 Mha, with a further rise to 195.0–231.2 Mha under the palm oil replacement scenarios (Table S2). The identified land is predominantly situated in India, China, and Bangladesh, south (Bolivia, Brazil, and Venezuela); and central America (Cuba and Guatemala); Central Africa; Zambia; Tanzania; Mozambique and Madagascar, the USA, Indonesia, and Australia (see Figure S3). The primary apparent risk lies in food security, as a relevant portion of land currently allocated to annual crops results in a suitable and could potentially be impacted for future soybean oil production, with 40.7–63.9 Mha under the scenario with the current consumption rate and 75.1–94.3 Mha under the EAT‐Lancet scenario. Additionally, forest ecosystems and shrublands face a significant threat, as future soybean oil production might impact 31.3–48.3 Mha of land currently covered by forests and 26.6–26.8 Mha of shrublands under the scenario with the current consumption rate. Under the EAT‐Lancet scenario, these numbers increase to 59.8–80.6 and 29.5–34.9 Mha for forests and shrublands, respectively.

The total land required to satisfy the food palm oil and unsaturated oils demand in 2050 is estimated at 188–189 Mha under the current consumption rate scenario and 317 Mha under the EAT‐Lancet scenario and tends to increase with the growing percentage of palm oil replacement, ranging from 25% to 100%, resulting in 254 Mha to 385 Mha in the respective scenarios (Table 2). The land needed for palm oil remains approximately the same when assessed prioritizing higher yields or proximity to existing oil palm plantations, even though its geographical distribution changes.

Overall, there are 61.5–66 Mha of global forests facing the risk of deforestation to meet the future global food demand for palm oil and unsaturated oils if current consumption rates are projected to 2050 and 115–120 Mha under the future EAT‐Lancet scenario. The forests at risk of deforestation further increase to 66–88 Mha (or 70–88 Mha when oil palm plantations are distributed prioritizing areas by yields) and to 125–148 Mha (or 129–148 Mha when oil palm plantations are distributed prioritizing areas by yields) in the two consumption scenarios if the 25%–50%–100% food use of palm oil is progressively replaced by unsaturated oil (Table S2).

### 3.2 Potential GHG Emissions From Forest Conversion and Other Land Use Changes

We found that the world's vegetable oils requirement for food use in 2050 has a potential of GHG emissions of 621–666 Mt. CO_2_ per year if the current consumption rate is projected, due to the possible carbon stock losses from LUC of high carbon content areas, such as forests, peatlands, and perennial croplands converted for oil crop cultivation. Potential LUC GHG emissions rise to 1163–1110 Mt. CO_2_ per year if the EAT‐Lancet consumption rates are followed, meaning an additional 542–544 Mt. CO_2_ per year, that is, up to an 87% rise in LUC GHG emissions compared to the projected current consumption rate (Table 2).

Interestingly, prioritizing proximity to existing plantations for future oil palm distribution yields a reduction of 39%–42% in GHG emissions from LUC for palm oil production compared to selecting areas based on the potential for higher attainable yields (i.e., 71 compared to 116 Mt. CO_2_ per year in the current consumption rate scenario and 65 compared to 112 Mt. CO_2_ per year in the EAT‐Lancet consumption scenario), despite the total land requirement remaining nearly unchanged, with only a negligible increase of +5% or +3.6% observed when prioritizing proximal areas in the two consumption scenarios. This is attributed to the optimized geographical distribution that makes efficient use of areas already designated for palm oil in Southeast Asia (Figures 1b and 2b), consequently impacting fewer forested areas and resulting in lower carbon stock losses.

In the case of palm oil substitution with the other oils, the potential GHG emissions from LUC tend to increase with the growing percentage of replacement (from 25% to 100%) up to 872 Mt. CO_2_ per year if palm oil is completely replaced in the current consumption rate scenario and to 1525 Mt. CO_2_ per year in the EAT‐Lancet consumption scenario (Table 2). This is due to the fact that when palm oil is replaced by the other three unsaturated oils, a greater amount of land is required proportionally. This is because the oil yields of the other three oilseed crops are significantly lower, ranging from 0.6 to 0.8 tons of oil per hectare, compared to oil palm, which can yield up to 4 tons of oil per hectare (Chiriacò et al. 2024).

In general, although potential annual GHG emissions from LUC for oilseed crop expansion are calculated including both forests and perennial cropland conversion as well as peatland drainage, the latter two LUCs (i.e., perennial cropland conversion and peatland drainage) are found to have minimal significance. They represent a relatively small portion of the total affected area, accounting for 2%–6% and 0.1%–0.2%, respectively (Table S2), and contribute only about 3% and 0.3% of the total GHG emissions, respectively (Table S3).

### 3.3 Threat to Food Security and Indirect Land Use Change

Results also highlight that future oil crop distribution for food use occurs to some extent in areas that are currently used for other crops, potentially threatening food security and causing, in its turn, a possible indirect land use change (Villoria 2019) to reallocate crop areas and ensure adequate production levels. For example (Table S4), about 2.7 Mha currently allocated to wheat cultivation would become suitable for rapeseed and another 3 Mha for sunflower if the current food consumption rate is projected to the 2050 global population. Wheat potentially replaced for food oil production rises to 3.5 Mha to satisfy future world's rapeseed food oil demand plus 5 Mha for future sunflower food oil demands if the EAT‐Lancet recommendations are followed. Future sunflower food oil demand could also potentially threaten 4.6 Mha of current maize cultivations or 8.9 Mha to satisfy EAT‐Lancet recommendations. Soybean future distribution for food oil production would instead potentially affect 9 Mha of the existing global rice cultivation when the current soybean food oil consumption rate is projected to 2050, more than doubling to 22.1 Mha if the EAT‐Lancet recommendations are followed, followed by 3 or 5 Mha of legumes and 3 or 5.4 Mha of tuberous, under the two scenarios. Additionally, around 1.2 Mha of current rice cultivations would be affected by the future distribution of oil palm plantations for food use when suitable areas are prioritized based on the potential for higher attainable yields, or 2.1 Mha when the distribution is assessed by proximity to existing plantations. These results remain consistent under the two consumption scenarios.

In general, the potential threat to food security increases if 25% to 100% of palm oil food use is replaced by rapeseed, sunflower, and soybean oil. In the worst scenario, up to 4.7 Mha of areas currently allocated to wheat would become suitable for rapeseed and another 6 Mha for sunflower, which also could potentially affect 12.2 Mha of current maize cultivations. Soybean future distribution under 100% palm oil food use replacement would instead potentially affect up to 28.6 Mha of rice, 6.7 Mha of legumes, and 6.7 Mha of tubers, against a reduction of pressure in rice cultivations to a total of 0.6 Mha (with oil palm areas of higher attainable yields) or 1 Mha (with oil palm areas proximal to existing plantations).

## 4 Discussion

**FIGURE 4:**
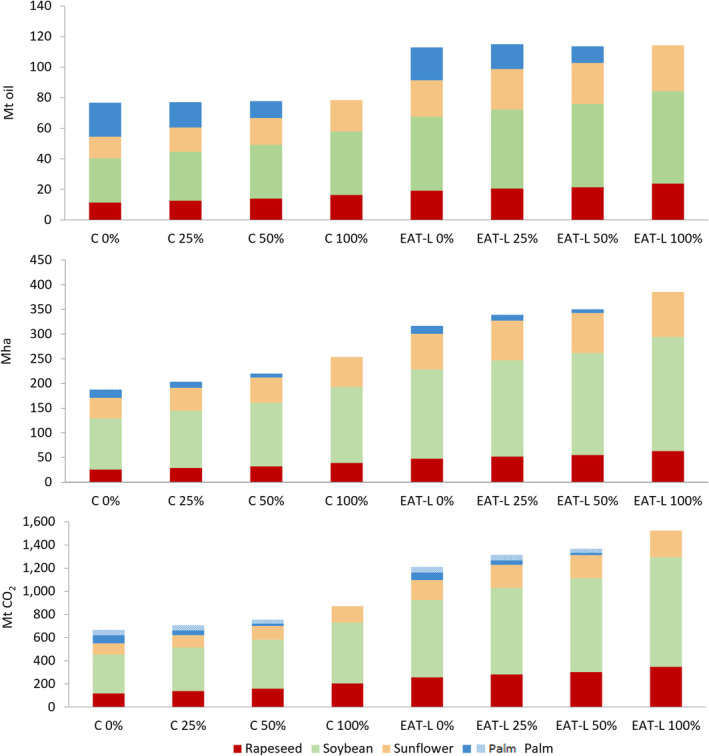
The needed amount of vegetable oils (Mt), land required for their cultivation (Mha), and potential associated GHG emissions from land use change (Mt CO_2_) to satisfy the global food oils demand in 2050 under the SPP2. Different scenarios are built projecting the current consumption rate (C) and following the EAT‐Lancet consumption (EAT‐L) and hypothesizing 0%, 25%, 50%, and 100% palm oil replacement with the three other oils (rapeseed—yellow bars, soybean—grey bars, sunflower—orange bars). Full blue bars represent palm oil when assessed prioritizing areas with higher yields, while blue‐dot bars complement the full blue bars when palm oil is assessed prioritizing proximity to existing plantations.

The total required land and the associated land use change GHG emissions increase across the palm oil replacement scenarios, up to +35% under the current consumption rate scenario and +22% under the EAT‐Lancet scenario (Figure 4). What clearly emerges is that the EAT‐Lancet palm and unsaturated oils consumption, combined with the projected global population growth, would result in an increase in the global production of vegetable oils for food use and in an increase of land required for their production. This expansion would potentially exacerbate the pressure on forests and the threat to global food security, entailing a possible rise of total GHG emissions from land use change, with 1163 Mt. CO_2_ per year in contrast to 621 Mt. CO_2_ per year if the current consumption rate were to be maintained. However, it is noteworthy that the complete diet reported by the EAT‐Lancet envisions an increase in the average global consumption of certain food items, such as unsaturated vegetable oils, together with fruits and legumes, while also foreseeing a decrease in the global consumption of other highly impactful food items, such as red meat, starchy vegetables, and eggs (Willett et al. 2019). As such, this dietary shift aimed at promoting a healthful and well‐balanced diet with optimal caloric intake will result in an overall 49% reduction of the projected global GHG emissions, allowing to limit the global food production to the boundary of sustainability at 5 Gt CO_2_eq per year (Willett et al. 2019). Furthermore, even more favorable environmental outcomes of the the EAT‐Lancet diet could be attained by halving food loss and waste, which currently account for approximately one‐third of total food production (Willett et al. 2019; Gatto and Chepeliev 2024). Therefore, in view of the overall reduction in GHG emissions envisaged by EAT‐Lancet planetary diet pattern, this paper highlights, however, a specific increase in land use and possible GHG emissions from deforestation solely attributable to the future food consumption of vegetable oils. Furthermore, our analysis does not include the predictable land use and associated GHG emissions stemming from oil crop cultivation for future livestock feed purposes, which, however, currently account for 0.5% of the total vegetable oil production and are expected to further decrease if the EAT‐Lancet diet is adopted globally and alternative by‐products are used (Sandström et al. 2022; Govoni et al. 2023), thus making the cropland associated with feed production available for other uses (Erb et al. 2016).

Another significant finding relies on the fact that replacing palm oil in food usage with other oils is never an advantageous solution, both because saturated and unsaturated oils have diverse properties and health effects and because both the total required land and the associated GHG emissions from land use change would arise. Specifically, projecting the current consumption rate to 2050, the total land required would increase by 35%, reaching 254 Mha if 100% of palm oil for food use were substituted with unsaturated oils, in contrast to the 189 Mha that would suffice if palm oil were not replaced. Consequently, an additional 26.5 Mha (or 22 Mha when oil palm plantations are distributed prioritizing areas by yields) of forests are at potential risk of deforestation, reaching 88 Mha if 100% of palm oil for food use were replaced, in contrast to 61.5 Mha (or 66 Mha when oil palm plantations are distributed prioritizing areas by yields) that are at risk if palm oil were not replaced for food use. Also, GHG emissions would increase by 41% up to 872 Mt. CO_2_ per year if 100% palm oil for food use were to be replaced, against 621 Mt. CO_2_ per year (or 666 Mt. CO_2_ per year when oil palm plantations are distributed prioritizing areas by yields) that would be emitted in case of no palm oil replacement. A similar trend with higher impacts is observed projecting the EAT‐Lancet rate. In this case, the total land required would increase by 21%, reaching 385 Mha if 100% of palm oil for food use were substituted with unsaturated oils, reaching up to 148 Mha forests at potential risk of deforestation, and GHG emissions would increase up to 1525 Mt. CO_2_ per year.

Similar evidence was reported by Parsons et al. (2020), who highlighted that substituting palm oil with other vegetable oils, such as rapeseed, sunflower, coconut oils, and shea butter, is environmentally and economically disadvantageous on a large scale, while ensuring sustainability of palm oil production is the only realistic approach to reduce the environmental impact. In fact, increasing land use efficiency rather than fostering an uncontrolled land use expansion, in particular for agricultural purposes, is a necessary strategy to preserve forests and their functions (Lambin and Meyfroidt 2011).

Actions to contrast deforestation are being taken at the global and national levels. 145 countries, representing more than 90% of the world's forests, signed the Global Deforestation Pledge proposed at the 26th Conference of Parties (COP26) of the United Nations Framework Convention on Climate Change (UNFCCC) in Glasgow in 2021, with the aim to halt and reverse forest loss and land degradation by 2030 while delivering sustainable development and promoting an inclusive rural transformation, so to design effective and equitable zero‐deforestation supply chains (Grabs et al. 2021). Moreover, many countries at the global level have committed themselves to specific national policy pledges to protect their forests and contrast domestic deforestation. Therefore, the actual risk of deforestation of areas suitable for the future distribution of oilseed crops can be more or less significant in each country depending on the type of national forest protection policy and level of commitment currently adopted and eventually maintained or fostered in the future.

However, the increasing demand for vegetable oils has raised awareness regarding the need to engage in more sustainable production methods. Specific certification schemes and protocols have been developed and adopted, in particular for palm oil, outlining environmental and socio‐economic principles and criteria for sustainable production and preserving existing forests and peatlands (Yaap and Paoli 2014; McInnes 2017; Schlösser and Walter 2020; Chiriacò et al. 2022). According to the Roundtable on Sustainable Palm Oil (RSPO) data (RSPO 2021)—one of the most comprehensive certification schemes (Yaap and Paoli 2014; McInnes 2017; Schlösser and Walter 2020)—currently 4.8 Mha out of the 28 Mha of oil palm plantations worldwide are certified as sustainable, meaning that they do not come from any agricultural‐driven deforestation, accounting for a total of 15 Mt. palm oil, which represents about the of its global production. In general, a relevant portion of GHG emissions potentially arising from the use of all vegetable oils could be cut if deforestation‐free is ensured for their crops' expansion, particularly in those countries where deforestation usually occurs. Thus, considering the growing relevance of certification schemes and protocols for sustainable and deforestation‐free supply chains (Yaap and Paoli 2014; McInnes 2017; Schlösser and Walter 2020; Chiriacò et al. 2022), especially in the case of palm oil (Afriyanti et al. 2016; Lam et al. 2019; Meijaard et al. 2020), it must be desirable that the attention to sustainable production would be paid to all vegetable oils, so to avoid the conversion of lands with high biodiversity (Fleiss et al. 2023; Gilroy 2023) and carbon content, such as forests, for any potential oilseed crop expansion, particularly in countries where deforestation is likely to occur or biodiversity is not adequately preserved. Potentials for such sustainable oil production while preserving biodiversity (Srinivasan et al. 2021; Strona 2021) or even optimizing ecological outcomes such as biodiversity, above‐ground carbon storage, and nutrient cycling if landscapes are properly planned (Bicknell et al. 2023; Rojas‐Castillo et al. 2023; Runting and Wells 2023) have been highlighted both for palm oil as well as for other vegetable oils (Bai et al. 2021).

## Author Contributions

**Maria Vincenza Chiriacò:** conceptualization, data curation, formal analysis, funding acquisition, investigation, methodology, project administration, resources, supervision, validation, visualization, writing – original draft, writing – review and editing. **Nikolas Galli:** conceptualization, data curation, formal analysis, methodology, visualization, writing – original draft, writing – review and editing. **Melissa Latella:** conceptualization, data curation. **Maria Cristina Rulli:** conceptualization, investigation, methodology, supervision, validation, writing – original draft, writing – review and editing.

## Conflicts of Interest

The authors declare no conflicts of interest.

## Supporting information


**Data S1.**.

**Figure S1.**.

**Table S1.**.

## Data Availability

The data and code that support the findings of this study are openly available in Zenodo at http://doi.org/10.5281/zenodo.14720442 and http://doi.org/10.5281/zenodo.14766767, respectively. The crop‐specific suitability as well as the crop‐specific attainable yield were obtained from the Global Agro‐Ecological Zones Data Portal at https://doi.org/10.4060/cb4744en. Annual and perennial crop‐specific areas were obtained from SPAM 2010 v2.0 Global Data at https://doi.org/10.7910/DVN/SWPENT. Area of other land‐uses were obtained from the ESA‐CCI landcover maps at https://doi.org/10.5194/essd‐15‐1465‐2023.
