# Supplementary material for: Pressure on Global Forests: Implications of Rising Vegetable Oils Consumption Under the EAT‐Lancet Diet
Source: Glob Chang Biol. 2025 Feb 20;31(2):e70077. doi: 10.1111/gcb.70077 (PMC11840662; doi:10.1111/gcb.70077)
Supplement: Supplementary file 1 — Data S1.. [file GCB-31-e70077-s002.docx]

**Equations to calculate oil production**

The equations utilize attainable crop yields expressed as seed yield (FAO, 2021) and apply the oil/seed ratio of 0.20 for soybean (Dijkstra, 2016) and 0.35 for rapeseed (Wei et al., 2008) and sunflower (Le Clef & Kemper, 2015). For palm oil, the attainable oil yield is directly provided.

Furthermore, for soybean, which commonly yields products beyond oil (such as flour, lecithin, soymilk, beans, and others), the equation includes the global average ratio of seeds designated for oil production out of the total seed yield (84%), as sourced from FAOSTAT (2023).

Additionally, the equations adjust the total attainable oil production allocating the portion designated for food use only, proportionally to the global average share of oil allocated for food use over the total production, being 25% for palm oil, 38% for rapeseed oil, 41% for soybean oil, and 61% for sunflower oil (fifth column in Table S1) as derived by FAOSTAT (2023).

(Eq. 1) ${PO}_{(f)}=P\_oy \times0.25$

(Eq. 2) ${RsO}_{(f)}=Rs\_sy \times0.35 \times0.38$

(Eq. 3) ${SbO}_{(f)}=Sb\_sy \times0.20 \times0.84 \times0.41$

(Eq. 4) ${SfO}_{(f)}=Sf\_sy \times0.35 \times0.61$

where:

*Po_(f)_* is palm oil production for food use.

*Rso_(f)_* is rapeseed oil production for food use.

*Sbo_(f)_* is soybean oil production for food use.

*Sfo_(f)_* is sunflower oil production for food use.

*P_oy* is palm oil attainable yield.

*Rs_sy* is rapeseed attainable seed yield.

*Sb_sy* is soybean attainable seed yield.

*Sf_sy* is sunflower attainable seed yield.

# Land use change data quality assessment

Some errors may arise due to the fact that we use different types of land-cover datasets coming from different sources. For instance, in some cases, different datasets classify the same area in different ways, so that it gets counted more than once for the allocation, leading to available areas in a pixel that are slightly higher than the total area itself. However, this happens less than 2% of the pixels. Therefore, the error in this sense can be considered negligible.
