# Supplementary material for: Pressure on Global Forests: Implications of Rising Vegetable Oils Consumption Under the EAT‐Lancet Diet
Source: Glob Chang Biol. 2025 Feb 20;31(2):e70077. doi: 10.1111/gcb.70077 (PMC11840662; doi:10.1111/gcb.70077)
Supplement: Supplementary file 2 — Figure S1.. [file GCB-31-e70077-s001.docx]

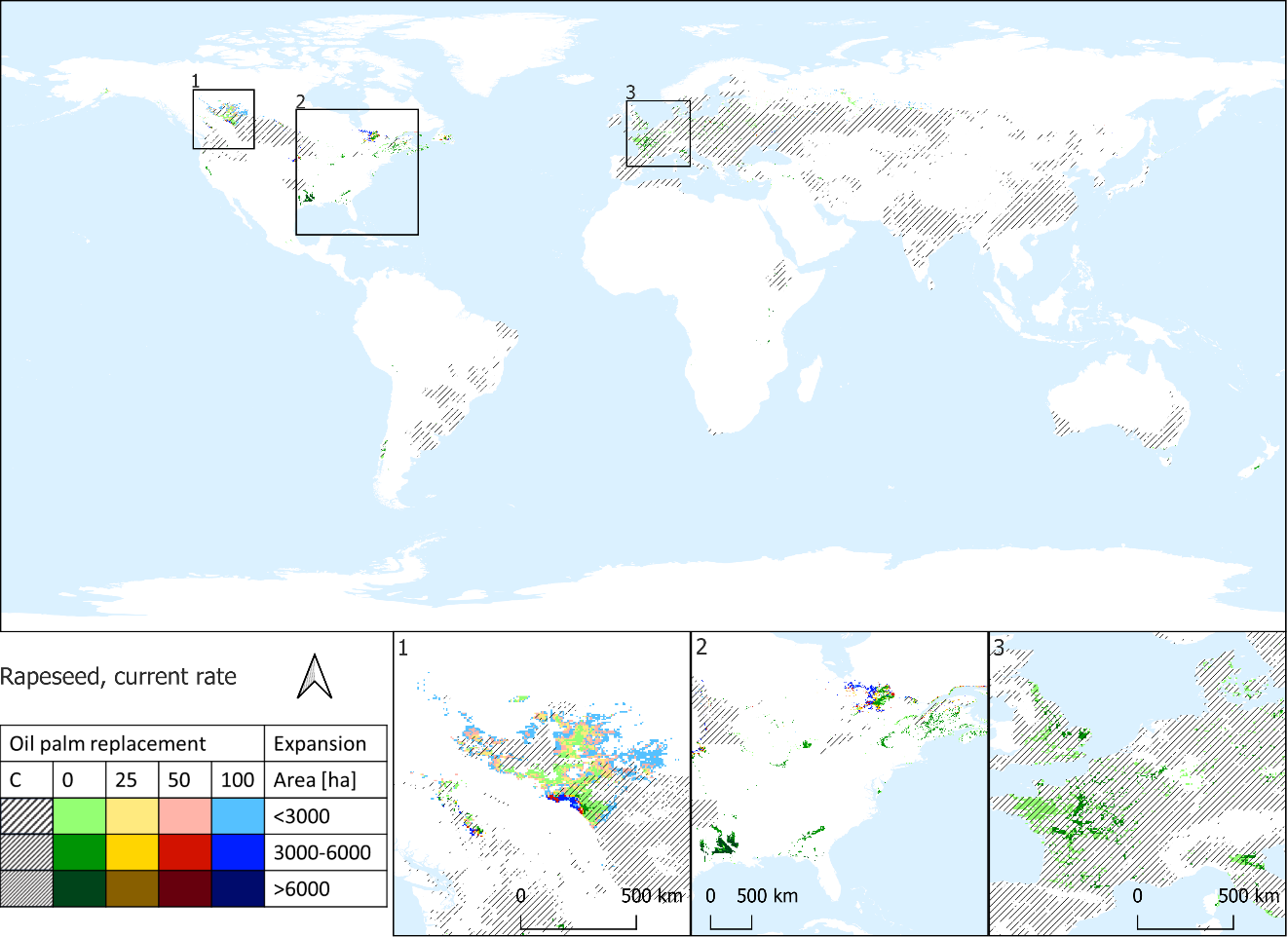


b)

a)


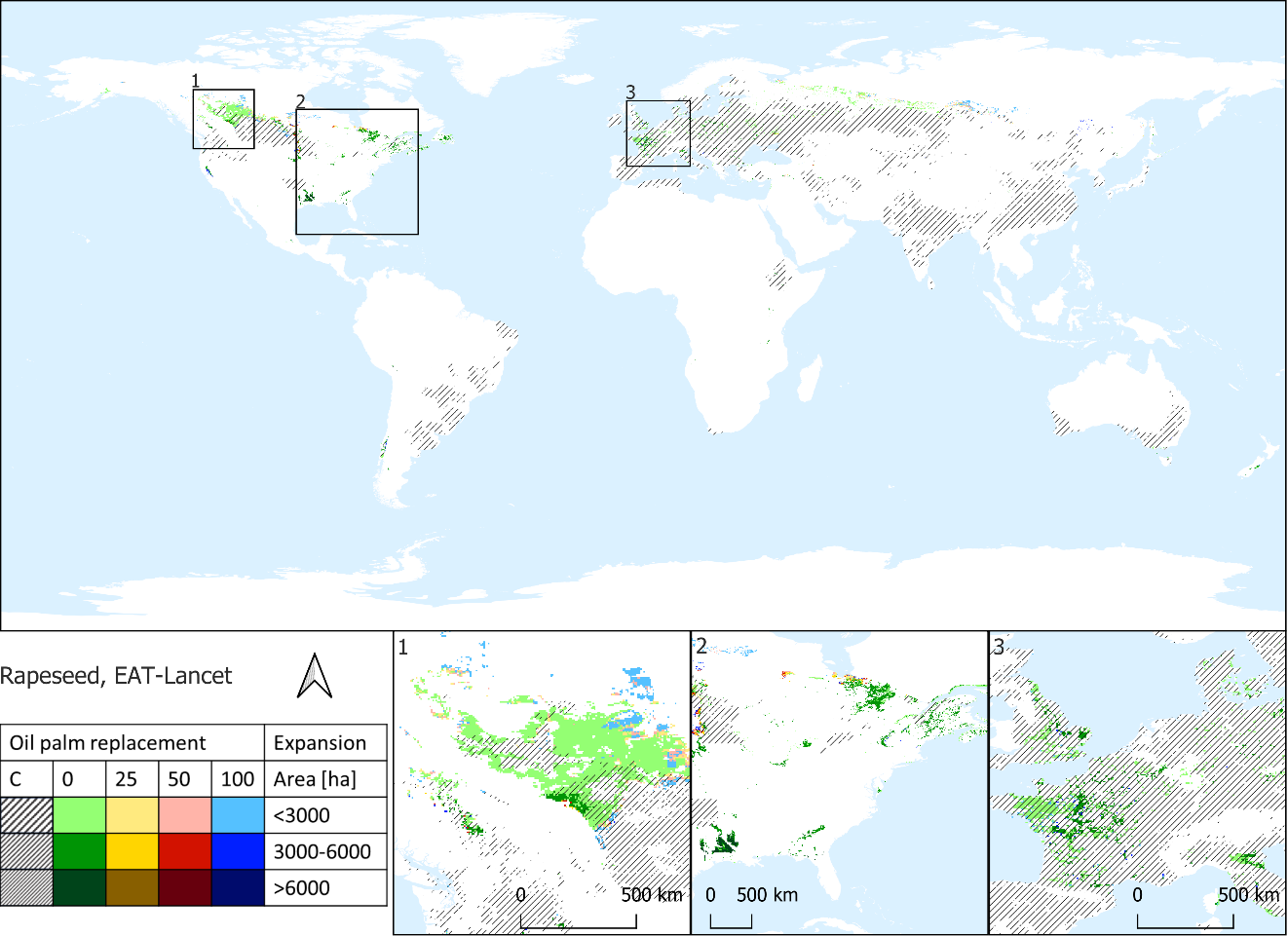


**Figure 1S. Potential future distribution of rapeseed crops for food oil use in 2050 under the 'current consumption rate scenario' (Fig. 1Sa) and 'EAT-Lancet recommended rate scenario' (Fig. 1Sb). Green pixels indicate areas suitable to cover the no palm oil replacement scenario; yellow pixels indicate areas suitable to complement the green areas to satisfy the 25% palm oil replacement scenario; red pixels indicate areas suitable to complement green and yellow areas to satisfy the 50% palm oil replacement scenario; blue pixels indicate areas suitable to complement green, yellow and red areas to satisfy the 100% palm oil replacement scenario. Each colored pixel is represented with three cover intensities (<3 kha, 3-6 kha, >6 kha).**


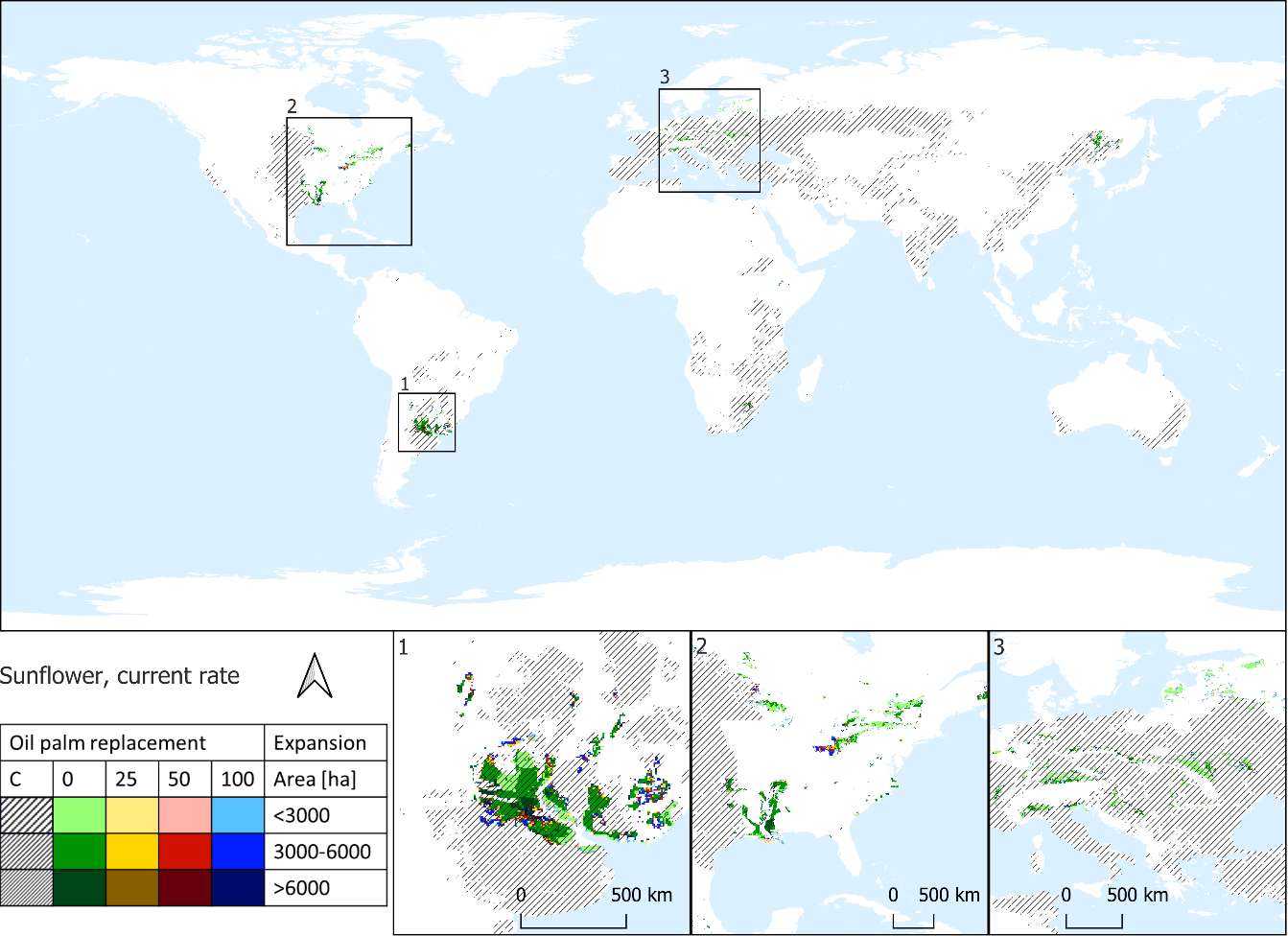


b)

a)


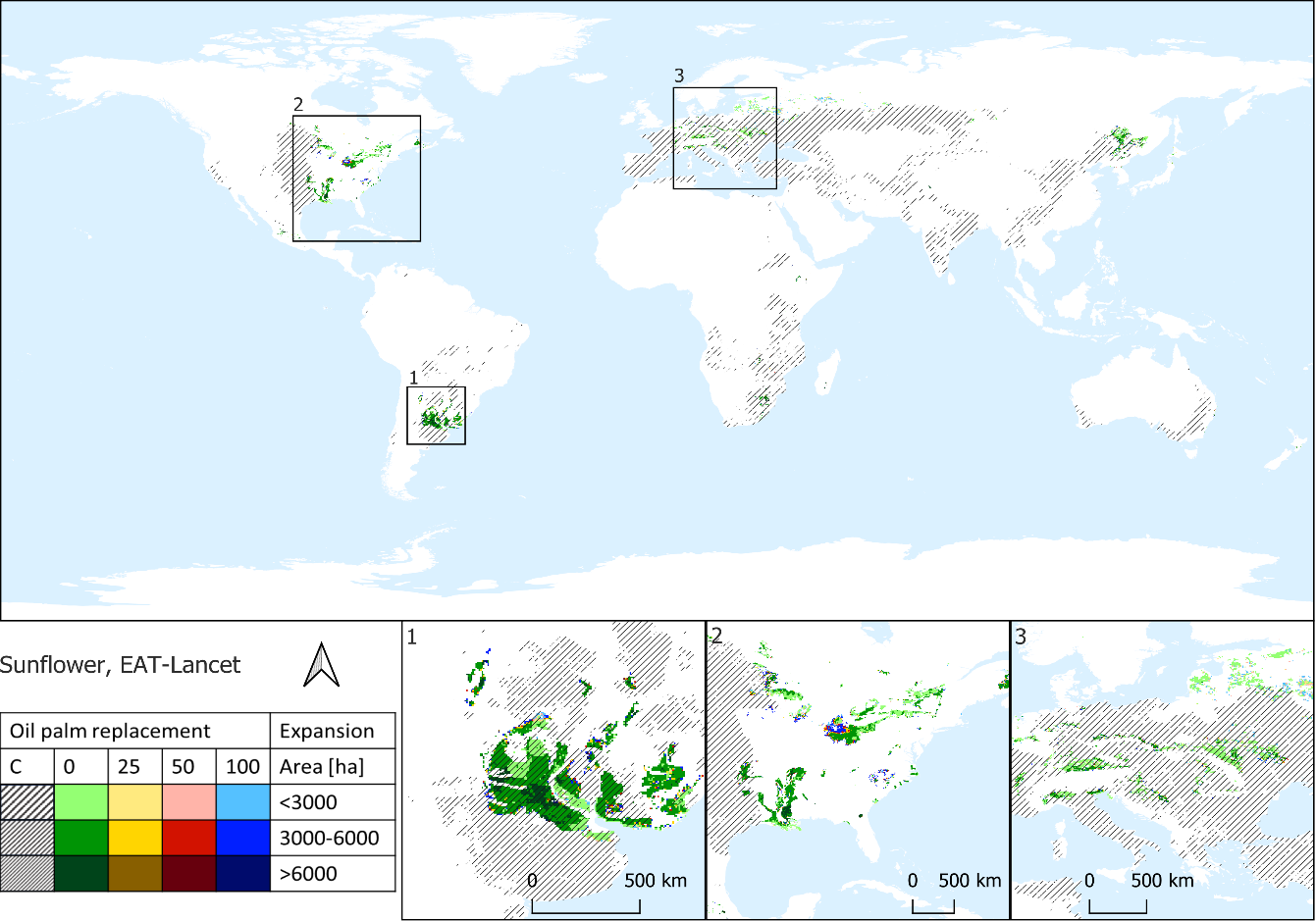


**Figure 2S. Potential future distribution of sunflower crops for food oil use in 2050 under the 'current consumption rate scenario' (Fig. 2Sa) and 'EAT-Lancet recommended rate scenario' (Fig. 2Sb). Green pixels indicate areas suitable to cover the no palm oil replacement scenario; yellow pixels indicate areas suitable to complement the green areas to satisfy the 25% palm oil replacement scenario; red pixels indicate areas suitable to complement green and yellow areas to satisfy the 50% palm oil replacement scenario; blue pixels indicate areas suitable to complement green, yellow and red areas to satisfy the 100% palm oil replacement scenario. Each colored pixel is represented with three cover intensities (<3 kha, 3-6 kha, >6 kha).**


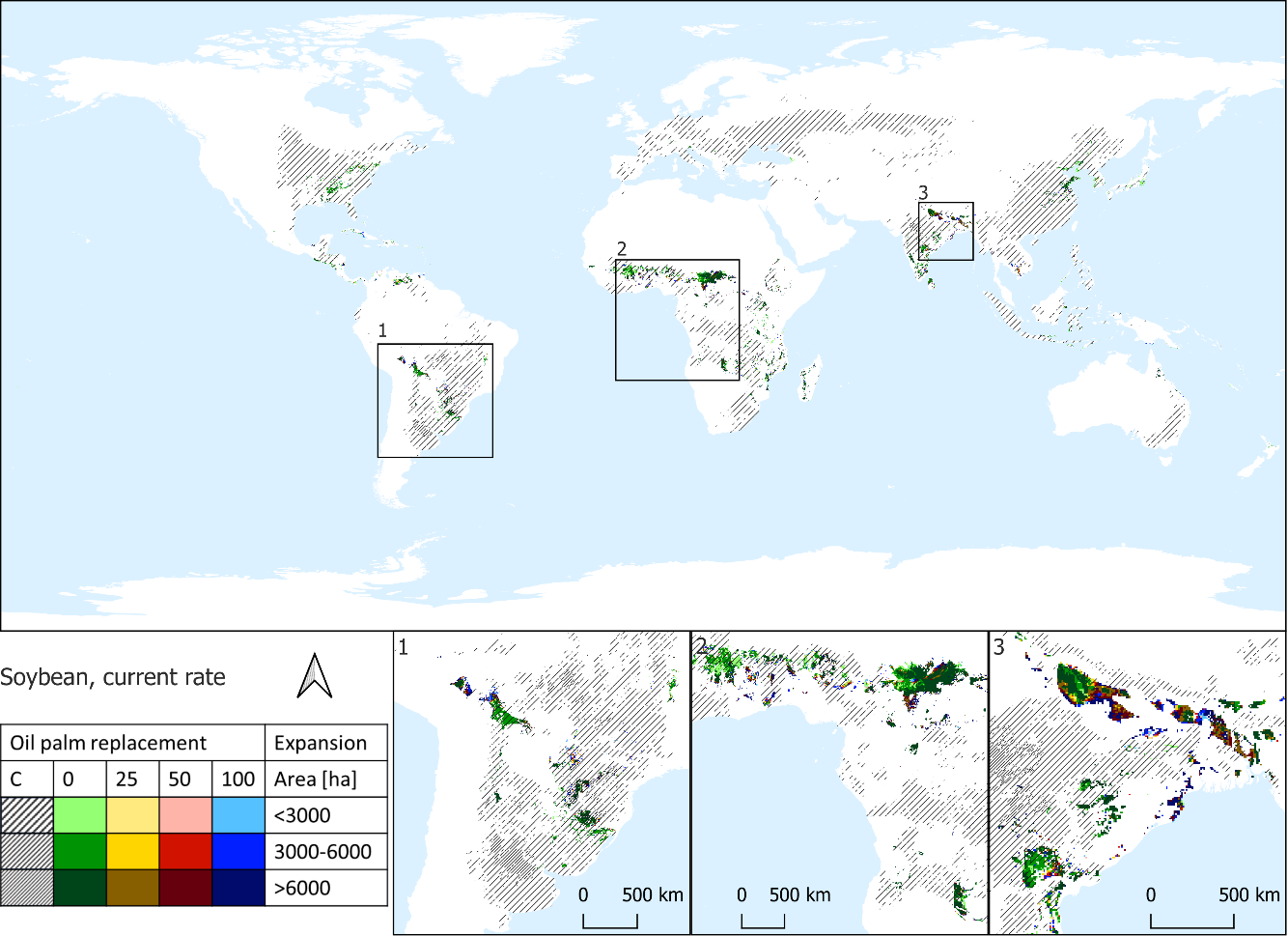


b)

a)


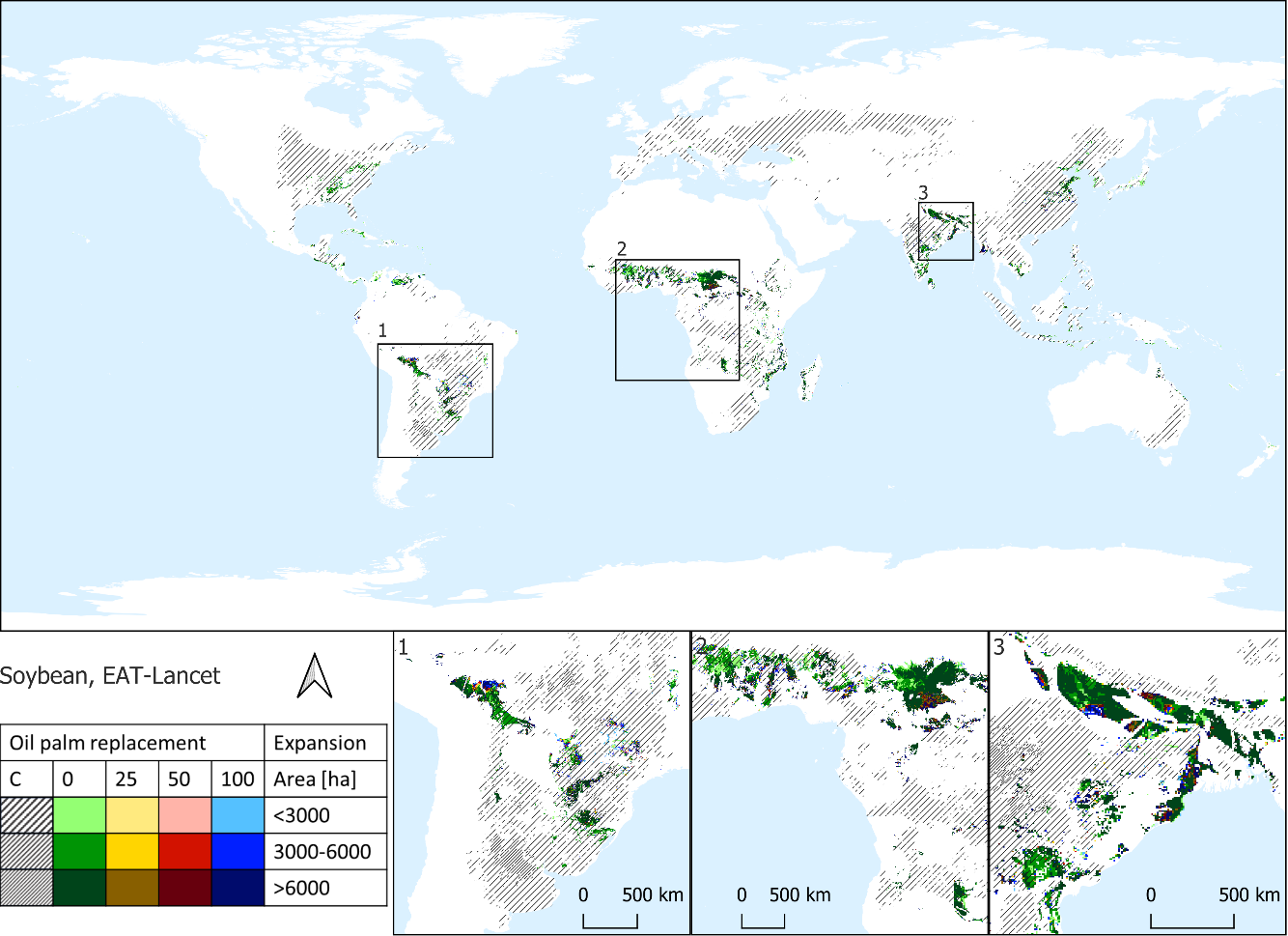


**Figure 3S. Potential future distribution of sunflower crops for food oil use in 2050 under the 'current consumption rate scenario' (Fig. 3Sa) and 'EAT-Lancet recommended rate scenario' (Fig. 3Sb). Green pixels indicate areas suitable to cover the no palm oil replacement scenario; yellow pixels indicate areas suitable to complement the green areas to satisfy the 25% palm oil replacement scenario; red pixels indicate areas suitable to complement green and yellow areas to satisfy the 50% palm oil replacement scenario; blue pixels indicate areas suitable to complement green, yellow and red areas to satisfy the 100% palm oil replacement scenario. Each colored pixel is represented with three cover intensities (<3 kha, 3-6 kha, >6 kha).**
