# Supplementary material for: Pressure on Global Forests: Implications of Rising Vegetable Oils Consumption Under the EAT‐Lancet Diet
Source: Glob Chang Biol. 2025 Feb 20;31(2):e70077. doi: 10.1111/gcb.70077 (PMC11840662; doi:10.1111/gcb.70077)
Supplement: Supplementary file 3 — Table S1.. [file GCB-31-e70077-s003.docx]

**Table S1. Global vegetable oils data, average 2018-2020 (FAOSTAT, 2023).**

| **Oil** | **Harvested area**  **(kha)** | **Total oil production (kt)** | **Oil food consumption (kt)** | **Oil food use over total production** | **Share of total main oils’ production** |
| --- | --- | --- | --- | --- | --- |
| Palm | 28,191 | 73,415 | 18,368 | 25% | *93%* |
| Palm kernel |  | 7,934 | 1,512 | 19% | *7%* |
| Rapeseed | 35,370 | 24,945 | 9,549 | 38% | 21% |
| Soybean | 124,133 | 58,338 | 24,089 | 41% | 53% |
| Sunflower | 27,280 | 19,657 | 11,919 | 61% | 26% |
| *Total palm and main unsaturated oils* | *214,974* | *184,289* | *65,437* | *36%* |  |
| Others | 114,591 | 39,647 | 21,488 | 54% |  |
| **Totals** | **329,565** | **223,936** | **86,925** | 39% |  |

**Table S2. Available area per land use type for future oilseed crops distribution to fulfill the global food oil demand under the current and recommended EAT-Lancet consumption rate scenarios and with different Palm Oil (PO) replacement scenarios. Suitability classes: 1. very high, 2. high, 3. good, 4. medium, 5. moderate, 6. marginal, 7. very marginal, 8. not suitable (FAO, 2021).**

| **Oil** | **Consump. Scenario** | **PO repl. Scenario** | **Suit. class** | | **Minimum yield level**  **(kg/ha)** | **Total oil production (M tons)** | **Oil for food use (M tons)** | **Total area for food use**  **(kha)** | | **Annual Cropland (kha)** | | **Perennial Cropland (kha)** | | **Grassland**  **(kha)** | | **Shrubland (kha)** | | **Forests (kha)** | | **Bare (kha)** | | **Peatlands (kha)** |
| --- | --- | --- | --- | --- | --- | --- | --- | --- | --- | --- | --- | --- | --- | --- | --- | --- | --- | --- | --- | --- | --- | --- |
| Palm  (by yield) | Current | 0% | | 2 | 1,293.25 | 88.34 | 22.08 | 16,631 | 2,070 | | 2,267 | | 955 | | 68 | | 10,927 | | 344 | | 104 | |
|  |  | 25% | | 2 | 1,306.25 | 66.26 | 16.57 | 12,383 | 1,438 | | 1,617 | | 786 | | 48 | | 8,238 | | 257 | | 80 | |
|  |  | 50% | | 2 | 1,318.75 | 44.15 | 11.04 | 8,170 | 1,041 | | 1,194 | | 592 | | 37 | | 5,130 | | 177 | | 50 | |
|  |  | 100% | |  | - |  |  | 0 | 0 | | 0 | | 0 | | 0 | | 0 | | 0 | | 0 | |
|  | EAT-Lancet | 0% | | 2 | 1,294.75 | 85.70 | 21.42 | 16,121 | 1,993 | | 2,195 | | 942 | | 65 | | 10,592 | | 333 | | 101 | |
|  |  | 25% | | 2 | 1,307.25 | 64.37 | 16.09 | 12,021 | 1,387 | | 1,573 | | 746 | | 48 | | 8,020 | | 249 | | 77 | |
|  |  | 50% | | 2 | 1,319.50 | 43.14 | 10.79 | 7,979 | 1,022 | | 1,179 | | 562 | | 37 | | 5,004 | | 175 | | 49 | |
|  |  | 100% | |  | - |  |  | 0 | 0 | | 0 | | 0 | | 0 | | 0 | | 0 | | 0 | |
| Palm  (by area) | Current | 0% | | 2 | 0.6* | 88.88 | 22.22 | 17,456 | 4,489 | | 5,448 | | 551 | | 48 | | 6,520 | | 399 | | 88 | |
|  |  | 25% | | 2 | 3.2* | 66.14 | 16.53 | 12,976 | 3,626 | | 5,049 | | 319 | | 25 | | 3,777 | | 181 | | 65 | |
|  |  | 50% | | 2 | 9.0* | 44.18 | 11.05 | 8,674 | 2,411 | | 4,222 | | 182 | | 11 | | 1,793 | | 54 | | 38 | |
|  |  | 100% | |  | - | - | - | 0 | 0 | | 0 | | 0 | | 0 | | 0 | | 0 | | 0 | |
|  | EAT-Lancet | 0% | | 2 | 0.9* | 85.09 | 21.27 | 16,709 | 4,368 | | 5,413 | | 491 | | 47 | | 6,015 | | 375 | | 85 | |
|  |  | 25% | | 2 | 3.9* | 63.46 | 15.86 | 12,449 | 3,535 | | 4,982 | | 314 | | 23 | | 3,443 | | 153 | | 62 | |
|  |  | 50% | | 2 | 9.7* | 42.24 | 10.56 | 8,296 | 2,290 | | 4,110 | | 173 | | 6 | | 1,669 | | 47 | | 36 | |
|  |  | 100% | |  | - | - | - | 0 | 0 | | 0 | | 0 | | 0 | | 0 | | 0 | | 0 | |
| Rapeseed | Current | 0% | | 1 | 387.56 | 85.94 | 11.43 | 25,782 | 8,312 | | 473 | | 2,887 | | 757 | | 13,115 | | 237 | | 41 | |
|  |  | 25% | | 1 | 375.86 | 95.35 | 12.69 | 29,063 | 8,861 | | 484 | | 2,921 | | 963 | | 15,576 | | 258 | | 57 | |
|  |  | 50% | | 1 | 365.09 | 104.72 | 13.93 | 32,427 | 9,291 | | 487 | | 2,951 | | 1,191 | | 18,224 | | 283 | | 76 | |
|  |  | 100% | | 1 | 342.34 | 123.46 | 16.42 | 39,471 | 10,294 | | 494 | | 3,080 | | 1,524 | | 23,749 | | 331 | | 126 | |
|  | EAT-Lancet | 0% | | 1 | 316.81 | 143.67 | 19.11 | 47,630 | 11,707 | | 501 | | 3,256 | | 1,716 | | 30,066 | | 384 | | 212 | |
|  |  | 25% | | 1 | 303.77 | 153.90 | 20.47 | 52,012 | 12,585 | | 504 | | 3,324 | | 1,813 | | 33,372 | | 415 | | 255 | |
|  |  | 50% | | 1 | 291.14 | 161.55 | 21.49 | 55,431 | 13,353 | | 505 | | 3,365 | | 1,865 | | 35,905 | | 438 | | 283 | |
|  |  | 100% | | 2 | 428.26 | 179.48 | 23.87 | 63,371 | 14,976 | | 619 | | 3,656 | | 2,022 | | 41,591 | | 506 | | 329 | |
| Soybean | Current | 0% | | 2 | 257.75 | 419.68 | 28.91 | 103,481 | 40,760 | | 2,386 | | 8,137 | | 20,662 | | 31,257 | | 279 | | 53 | |
|  |  | 25% | | 2 | 255.20 | 465.23 | 32.05 | 115,718 | 46,866 | | 2,679 | | 8,749 | | 22,433 | | 34,703 | | 289 | | 61 | |
|  |  | 50% | | 2 | 252.79 | 512.02 | 35.27 | 128,407 | 52,815 | | 2,956 | | 9,328 | | 24,082 | | 38,923 | | 302 | | 69 | |
|  |  | 100% | | 2 | 248.38 | 602.60 | 41.51 | 153,316 | 63,889 | | 3,539 | | 10,451 | | 26,794 | | 48,289 | | 353 | | 85 | |
|  | EAT-Lancet | 0% | | 2 | 244.46 | 702.65 | 48.40 | 181,290 | 75,143 | | 4,282 | | 12,126 | | 29,537 | | 59,803 | | 400 | | 98 | |
|  |  | 25% | | 2 | 242.60 | 751.22 | 51.74 | 195,028 | 80,138 | | 4,601 | | 13,170 | | 31,050 | | 65,648 | | 420 | | 103 | |
|  |  | 50% | | 2 | 241.15 | 790.49 | 54.45 | 206,213 | 84,403 | | 4,901 | | 13,982 | | 32,152 | | 70,344 | | 432 | | 108 | |
|  |  | 100% | | 2 | 237.98 | 877.41 | 60.43 | 231,204 | 94,311 | | 5,509 | | 15,451 | | 34,871 | | 80,595 | | 466 | | 123 | |
| Sunflower | Current | 0% | | 1 | 931.07 | 66.64 | 14.23 | 41,833 | 23,686 | | 350 | | 6,146 | | 893 | | 10,654 | | 104 | | 31 | |
|  |  | 25% | | 1 | 925.52 | 74.16 | 15.83 | 46,532 | 26,316 | | 384 | | 6,941 | | 1,075 | | 11,705 | | 112 | | 35 | |
|  |  | 50% | | 1 | 920.19 | 81.68 | 17.44 | 51,431 | 29,219 | | 431 | | 7,616 | | 1,231 | | 12,816 | | 118 | | 39 | |
|  |  | 100% | | 1 | 910.79 | 96.22 | 20.54 | 61,024 | 34,110 | | 514 | | 8,935 | | 1,604 | | 15,726 | | 136 | | 48 | |
|  | EAT-Lancet | 0% | | 1 | 902.25 | 112.07 | 23.93 | 71,719 | 39,417 | | 597 | | 10,328 | | 1,940 | | 19,288 | | 149 | | 59 | |
|  |  | 25% | | 1 | 895.85 | 124.76 | 26.64 | 80,376 | 43,420 | | 659 | | 11,517 | | 2,233 | | 22,386 | | 160 | | 69 | |
|  |  | 50% | | 1 | 895.42 | 125.73 | 26.84 | 81,029 | 43,762 | | 664 | | 11,612 | | 2,253 | | 22,576 | | 161 | | 69 | |
|  |  | 100% | | 1 | 889.01 | 140.22 | 29.94 | 90,888 | 48,556 | | 722 | | 12,859 | | 2,734 | | 25,841 | | 175 | | 79 | |

- % pixel area covered by oil palm

**Table S3. GHG emissions from LUC (Mt CO_2_eq)**

| **Oil crop** | **Affected land use** | **Current consumption rate** | | | | **EAT-Lancet recommended rate** | | | | |
| --- | --- | --- | --- | --- | --- | --- | --- | --- | --- | --- |
|  |  | **0%** | **25%** | **50%** | **100%** | | **0%** | **25%** | **50%** | **100%** |
| Rapeseed | Forests | 115.8 | 136.1 | 157.8 | 202.9 | | 253.2 | 279.2 | 299.1 | 344.4 |
|  | Peatlands | 0.17 | 0.23 | 0.30 | 0.46 | | 0.71 | 0.82 | 0.88 | 0.98 |
|  | Orchards | 2.42 | 2.52 | 2.55 | 2.61 | | 2.67 | 2.70 | 2.70 | 2.95 |
|  | **Total** | **118** | **139** | **161** | **206** | | **257** | **283** | **303** | **348** |
| Soybean | Forests | 323.6 | 363.2 | 410.3 | 509.7 | | 651.5 | 729.5 | 790.8 | 924.7 |
|  | Peatlands | 0.4 | 0.4 | 0.5 | 0.6 | | 0.7 | 0.7 | 0.8 | 0.9 |
|  | Orchards | 10.3 | 11.3 | 12.2 | 13.9 | | 16.2 | 17.2 | 18.1 | 21.0 |
|  | **Total** | **334** | **375** | **423** | **524** | | **668** | **747** | **810** | **947** |
| Sunflower | Forests | 95.1 | 104.5 | 114.2 | 138.9 | | 168.6 | 195.4 | 196.9 | 225.6 |
|  | Peatlands | 0.11 | 0.13 | 0.14 | 0.17 | | 0.21 | 0.22 | 0.24 | 0.27 |
|  | Orchards | 2.21 | 2.41 | 2.64 | 3.06 | | 3.62 | 3.91 | 4.06 | 4.51 |
|  | **Total** | **97** | **107** | **117** | **142** | | **172** | **199** | **201** | **230** |
| Palm | Forests | 115 | 86.1 | 53.6 |  | | 111.2 | 83.8 | 52.3 |  |
|  | Peatlands | 0.8 | 0.6 | 0.4 |  | | 0.7 | 0.6 | 0.4 |  |
|  | Orchards | 0.2 | 0.1 | 0.1 |  | | 0.2 | 0.1 | 0.1 |  |
|  | **Total** | **116** | **87** | **54** |  | | **112** | **84** | **53** |  |
| Palm (aree) | Forests | 66.2 | 38.0 | 18.5 |  | | 61.1 | 34.7 | 17.2 |  |
|  | Peatlands | 0.6 | 0.5 | 0.3 |  | | 0.6 | 0.5 | 0.3 |  |
|  | Orchards | 3.8 | 3.0 | 2.3 |  | | 3.7 | 2.9 | 2.1 |  |
|  | **Total** | **71** | **41** | **21** |  | | **65** | **38** | **20** |  |
| **TOTAL (by yield)** | | **666** | **708** | **755** | **872** | | **1’210** | **1’314** | **1’366** | **1’525** |
| **TOTAL (by area)** | | **621** | **662** | **722** | **872** | | **1’163** | **1’268** | **1’333** | **1’525** |

**Table S4. Area harvested with annual and perennial crops potentially affected by the future oilseed crop distribution to fulfill the global food oil demand under the current and recommended EAT-Lancet consumption rate scenarios and with different Palm Oil (PO) replacement scenarios.**

| **Target crop** | **Consump. Scenario** | **PO repl. Scenario** | **Annual crops (kha)** | | | | | | | | | **Perennial crops (kha)** | |
| --- | --- | --- | --- | --- | --- | --- | --- | --- | --- | --- | --- | --- | --- |
|  |  |  | **Target crop** | **Rice** | **Wheat** | **Maize** | **Other cereals** | **Legumes** | **Tuberous** | | **Other annual crops** | **Target crop**  **(oil palm)** | **Other perennial crops** |
| Rapeseed | Current | 0% | 738 | 194 | 2,753 | 1,211 | 1,718 | 206 | 400 | 1,092 | | - | 473 |
|  |  | 25% | 787 | 196 | 2,858 | 1,325 | 1,841 | 220 | 424 | 1,210 | | - | 484 |
|  |  | 50% | 828 | 196 | 2,944 | 1,433 | 1,936 | 229 | 443 | 1,282 | | - | 487 |
|  |  | 100% | 897 | 196 | 3,075 | 1,588 | 2,063 | 241 | 467 | 1,769 | | - | 494 |
|  | EAT-Lancet | 0% | 1,169 | 200 | 3,451 | 1,819 | 2,342 | 273 | 515 | 1,938 | | - | 501 |
|  |  | 25% | 1,340 | 203 | 3,729 | 1,968 | 2,508 | 296 | 557 | 1,984 | | - | 504 |
|  |  | 50% | 1,500 | 203 | 3,915 | 2,069 | 2,610 | 315 | 570 | 2,172 | | - | 505 |
|  |  | 100% | 1,886 | 365 | 4,690 | 2,256 | 2,961 | 372 | 624 | 1,823 | | - | 619 |
| Soybean | Current | 0% | 3,816 | 9,016 | 3,980 | 6,429 | 4,315 | 3,008 | 3,088 | 7,108 | | - | 2,386 |
|  |  | 25% | 4,037 | 11,623 | 4,822 | 6,979 | 4,645 | 3,359 | 3,450 | 7,950 | | - | 2,679 |
|  |  | 50% | 4,282 | 14,003 | 5,716 | 7,545 | 5,004 | 3,703 | 3,840 | 8,722 | | - | 2,956 |
|  |  | 100% | 4,908 | 18,037 | 7,119 | 8,697 | 5,763 | 4,347 | 4,576 | 10,442 | | - | 3,539 |
|  | EAT-Lancet | 0% | 5,567 | 22,123 | 8,315 | 9,919 | 6,537 | 5,063 | 5,392 | 12,226 | | - | 4,282 |
|  |  | 25% | 5,932 | 23,762 | 8,780 | 10,466 | 6,925 | 5,434 | 5,779 | 13,060 | | - | 4,601 |
|  |  | 50% | 6,155 | 25,228 | 9,150 | 10,870 | 7,265 | 5,808 | 6,061 | 13,866 | | - | 4,901 |
|  |  | 100% | 6,787 | 28,590 | 9,958 | 12,031 | 7,986 | 6,676 | 6,774 | 15,510 | | - | 5,849 |
| Sunflower | Current | 0% | 215 | 1,138 | 2,962 | 4,622 | 1,640 | 233 | 629 | 12,245 | | - | 277 |
|  |  | 25% | 239 | 1,287 | 3,291 | 5,227 | 1,826 | 264 | 704 | 13,478 | | - | 307 |
|  |  | 50% | 269 | 1,458 | 3,629 | 5,945 | 2,035 | 295 | 795 | 14,793 | | - | 341 |
|  |  | 100% | 327 | 1,691 | 4,271 | 7,241 | 2,422 | 352 | 975 | 16,830 | | - | 397 |
|  | EAT-Lancet | 0% | 381 | 1,893 | 4,966 | 8,870 | 2,808 | 413 | 1,141 | 18,945 | | - | 478 |
|  |  | 25% | 423 | 2,032 | 5,406 | 10,258 | 3,114 | 460 | 1,275 | 20,453 | | - | 514 |
|  |  | 50% | 427 | 2,036 | 5,436 | 10,360 | 3,132 | 462 | 1,284 | 20,625 | | - | 538 |
|  |  | 100% | 473 | 2,198 | 5,919 | 12,189 | 3,482 | 512 | 1,434 | 22,350 | | - | 597 |
| Palm  (by yield) | Current | 0% | - | 1,233 | 0 | 139 | 0 | 5 | 60 | 3,051 | | 1,662 | 605 |
|  |  | 25% | - | 823 | 0 | 116 | 0 | 3 | 45 | 2,638 | | 1,182 | 436 |
|  |  | 50% | - | 625 | 0 | 96 | 0 | 2 | 34 | 1,655 | | 873 | 321 |
|  |  | 100% | - | - | - | - | - | - | - | - | | - | - |
|  | EAT-Lancet | 0% | - | 1,177 | 0 | 137 | 0 | 5 | 58 | 617 | | 1,598 | 598 |
|  |  | 25% | - | 814 | 0 | 116 | 0 | 3 | 45 | 409 | | 1,171 | 402 |
|  |  | 50% | - | 623 | 0 | 96 | 0 | 2 | 33 | 268 | | 867 | 312 |
|  |  | 100% | - | - | - | - | - | - | - | - | | - | - |
| Palm  (by area) | Current | 0% | - | 2,172 | 0 | 301 | 0 | 8 | 170 | 2,992 | | 4,204 | 1,244 |
|  |  | 25% | - | 1,657 | 0 | 189 | 0 | 6 | 119 | 2,558 | | 4,069 | 980 |
|  |  | 50% | - | 1,052 | 0 | 146 | 0 | 4 | 86 | 1,535 | | 3,478 | 743 |
|  |  | 100% | - | - | - | - | - | - | - | - | | - | - |
|  | EAT-Lancet | 0% | - | 2,121 | 0 | 264 | 0 | 8 | 158 | 1,817 | | 4,196 | 1,217 |
|  |  | 25% | - | 1,612 | 0 | 185 | 0 | 5 | 114 | 1,619 | | 4,033 | 949 |
|  |  | 50% | - | 1,011 | 0 | 143 | 0 | 3 | 85 | 1,048 | | 3,415 | 695 |
|  |  | 100% | - | - | - | - | - | - | - | - | | - | - |
